# Effectiveness of the *Let’s Move It* multi-level vocational school-based intervention on physical activity and sedentary behavior: a cluster randomized trial

**DOI:** 10.1093/abm/kaaf023

**Published:** 2025-05-27

**Authors:** Nelli Hankonen, Ari Haukkala, Minttu Palsola, Matti Toivo Juhani Heino, Reijo Sund, Kari Tokola, Pilvikki Absetz, Vera Araújo-Soares, Falko F Sniehotta, Katja Borodulin, Antti Uutela, Taru Lintunen, Tommi Vasankari

**Affiliations:** Faculty of Social Sciences, Tampere University, 33014 Tampere, Finland; Faculty of Social Sciences, University of Helsinki, 00014 Helsinki, Finland; Helsinki Collegium for Advanced Studies, University of Helsinki, Finland; Faculty of Social Sciences, Tampere University, 33014 Tampere, Finland; Faculty of Social Sciences, University of Helsinki, 00014 Helsinki, Finland; Faculty of Social Sciences, University of Helsinki, 00014 Helsinki, Finland; Institute of Clinical Medicine, University of Eastern Finland, 70211 Kuopio, Finland; The UKK Institute for Health Promotion Research, 33500 Tampere, Finland; Faculty of Social Sciences, Tampere University, 33014 Tampere, Finland; Centre for Preventive Medicine and Digital Health (CPD), Medical Faculty Mannheim, Heidelberg University, 68167 Mannheim, Germany; NIHR Policy Research Unit, Newcastle University, NE2 4AX Newcastle, United Kingdom; Age Institute, 00250 Helsinki, Finland; Department of Lifestyle and Participation, Finnish Institute for Health and Welfare (THL), 00271 Helsinki, Finland; Faculty of Sport and Health Sciences, University of Jyväskylä, 40014 Jyväskylä, Finland; The UKK Institute for Health Promotion Research, 33500 Tampere, Finland; Faculty of Medicine and Health Technology, Tampere University, 33014 Tampere, Finland

**Keywords:** physical activity, sedentary behavior, theory-based intervention, randomized trial, self-determination theory, reasoned action approach, self-regulation

## Abstract

**Background:**

Low levels of physical activity (PA), more prevalent among those with low education, require effective interventions. Fewer trials have tested interventions to decrease sedentary behavior (SB). No school-based interventions have shown lasting effects on PA or SB in vocational schools.

**Purpose:**

To examine whether the *Let’s Move It* intervention has effects on behavioral and clinical outcomes among vocational students after 2 and 14 months.

**Methods:**

A cluster randomized trial in 6 school units in vocational education in Finland (*N* = 1112) (mean age 18.5 years, range 15–46). The multi-component intervention targeted in-class activity opportunities (eg, teacher-led activity breaks, equipment in classrooms), and students’ motivation and self-regulation (eg, 6 group sessions, à 45–60 min, during the intensive intervention period of 2 months). Valid (≥ 4 days, ≥ 10 h/day) accelerometer data were obtained from 741 students at baseline, 521 (70.3%) at 2 months, and 406 (54.8%) at 14 months.

**Results:**

No evidence of a significant intervention effect on the co-primary outcomes (moderate-to-vigorous PA, SB, breaks in SB) was found. Participants in the intervention arm reduced their total daily SB time by 32 min (95% CI, −43.2 to −20.8) on weekdays, compared with the control arm’s reduction of 8.6 (95% CI, −19.5 to 2.3) and engaged in more accelerometer-measured light PA during school time. Few differences were found in secondary outcomes. The fidelity of intervention delivery was relatively good.

**Conclusions:**

This school-based intervention did not affect leisure-time activity. Despite a positive outcome on school-time light PA, more comprehensive or intensive environmental changes may be needed to meaningfully improve vocational students’ total activity.

## Teaser

Vocational school based intervention increased light-intensity physical activity, but had no effect on moderate-to-vigorous physical activity nor sedentary behavior

## Background

Physical activity (PA) is low and sedentary behavior (SB) is high in almost all populations,^1^ and especially among young people,^2–4^ those with lower socioeconomic status (SES),^5–7^ and females.^1,8^ Current physical activity recommendations underline the importance of increasing moderate-to-vigorous intensity physical activity (MVPA),^3^ decreasing total SB and introducing interruptions to SB (ie, breaks from sitting or lying down),^9–11^ while current evidence also emphasizes the health benefits of light physical activity.^9–12^ Interventions that effectively increase PA and reduce SB could therefore help to prevent non-communicable diseases^13^ and reduce health disparities.^7^

School-based interventions targeting PA and SB have been intensively studied in primary and (lower) secondary schools,^14,15^ with findings showing more effectiveness among girls (eg,^16^) but there is a dearth of high-quality PA intervention research in vocational education settings.^17^ Vocational schools are a particularly interesting setting for school-based PA intervention: they include a vulnerable group—low-SES individuals—just before entering work life, which requires work ability (see also^17^).

PA intervention research in vocational schools has been criticized for weak study quality and lack of long-term follow-ups.^17^ Similarly, randomized trials of school-based PA and SB interventions on the upper-secondary level have had methodological limitations such as low power, self-report measures of activity, and poor descriptions of intervention content.^18^ On average, school-based interventions overall have produced minimal effects on accelerometer-measured MVPA.^19^ Regardless, school-based interventions still hold promise^20^ due to their ability to reach large segments of the population where they spend most of their daytime hours.

Evidence on interventions in school settings seems to support the use of multiple strategies, as opposed to classroom-based or environmental strategies only.^21^ Interventions should thus incorporate several components, including environmental, classroom-based, and extracurricular activities. Still, to date, the majority of multi-component PA interventions include few components.^22^ Although PA and SB are separate behaviors, these constitute a continuum, and an increasing amount of interventions address both.^23^

We developed *Let’s Move It*, a whole school system intervention to increase PA and reduce SB, including various components targeting both the school, neighborhood as well as individual levels. Quantitative investigations noted that on average, intervention arm students self-reported having set PA goals, self-monitored their PA, motivated themselves more to engage in PA, and used other similar strategies more than the control arm students did,^24^ and showed some positive, yet short-lived, changes in SB-related cognitions.^25^ The intervention also taught teachers behavior change skills to provide more classroom activation for students.^26^ Unlike these process evaluations, the current paper reports the main outcome evaluation, that is, the effectiveness of the intervention. We examine the effects of the *Let’s Move It* intervention on changes in students’ PA and SB over 2 months and 14 months.

### Hypotheses

We test the hypothesis that the intervention is effective in increasing or maintaining PA and reducing SB, compared to standard curriculum, specifically in the following co-primary outcomes: MVPA, as measured by accelerometers and self-report, and SB as sedentary time and breaks in SB (as measured by accelerometry). We also evaluate the hypothesis that there is a significant difference in the change in MVPA, specifically among students with low or moderate levels of MVPA at baseline.

Secondary outcomes include, for example, other indicators of activity (eg, light PA, standing), body composition, self-reported fitness, and mental well-being.^27^ As accelerometry only encompasses 7 days at most on each measurement occasion, it may not be representative of a typical activity. Therefore, eventual changes in body composition (eg, lean mass) and self-reported fitness may better reflect sustained levels of activity over several months. Due to well-known differences between weekday and weekend activity,^28^ we also conduct additional exploratory analyses with weekdays and weekend days separately, as well as school time. Furthermore, we conducted secondary analyses on the effects of gender and dose received.

## Methods

### Study design and setting

This cluster randomized trial with study assessments conducted at baseline and 2 and 14 months after the baseline was conducted in 6 school units in Southern Finland in 2015-2017. The trial was reviewed by the ethical committee for gynecology and obstetrics, pediatrics, and psychiatry of The Hospital District of Helsinki and Uusimaa (367/13/03/03/2014) and registered (Trial registration number moved for masked review) when participant recruitment was ongoing. Detailed descriptions of the design and recruitment^27^ and intervention development^29^ have been published.

To determine the sample size, we used the power calculation approach by Eldridge and Kerry^30^ to define the design effect, by which a sample size derived for a difference in means would be inflated in a 2-level cluster randomized design. We assumed unequal cluster sizes (*M* = 17, *SD* = 3.83), derived from an internal pilot study. We aimed for at least 1100 participants at baseline, and details (eg, the intraclass correlation coefficient (ICCs used) are reported in the protocol.^27^ The spreadsheet with the syntax used for sample size calculations is publicly available.^31^

Vocational education and training (VET) in Finland is meant for both young people without upper secondary qualifications and for adults already in work life. Approximately half of each age cohort obtains a vocational degree, and half continue to high school and higher academic education. VET is organized mainly in institutions (on-the-job learning included) or as apprenticeship training. In an institutional setting, a qualification will usually take approximately 3 years to complete and is free of charge. A vocational qualification gives general eligibility for the university of applied science and university studies. The Ministry of Education and Culture prepares VET legislation and steers and supervises the sector. The Ministry also grants the education providers’ permits to provide VET.

### Participants and recruitment

**Figure 1.**
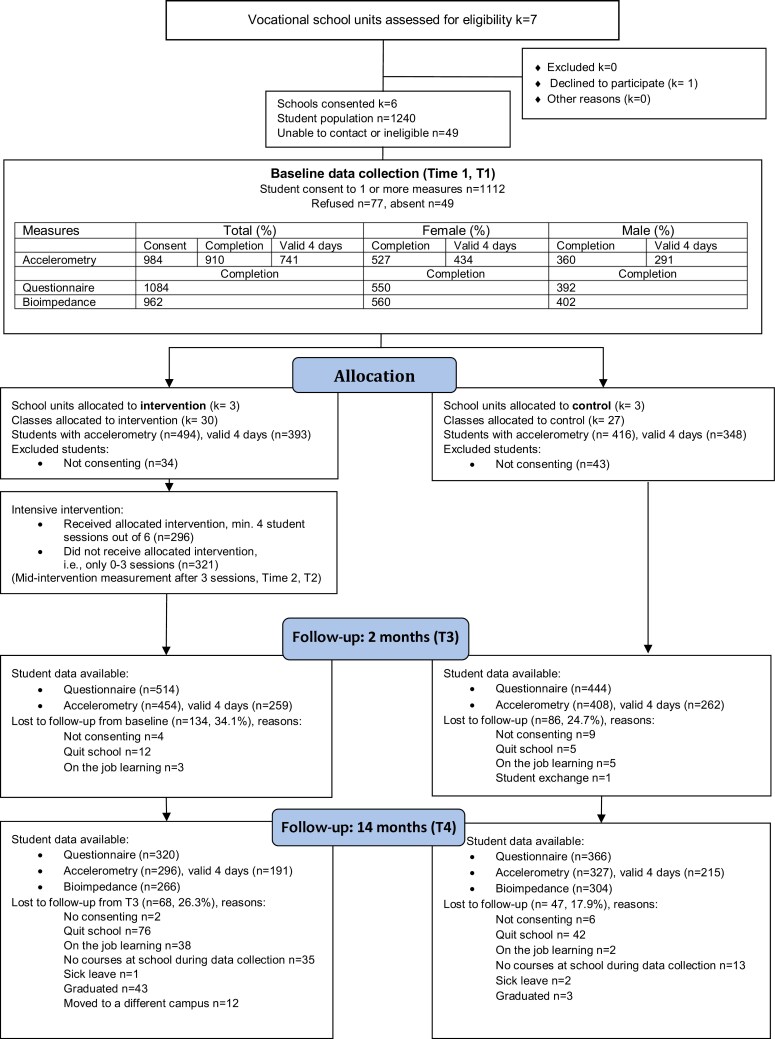
Flow diagram.

Figure 1 depicts the participant flow. Age ranged from 16 to 49 years, with the average age being 18.50. Only 16% (ie, 190 students) were 20 years or older. On average, the sample was relatively balanced between males and females (43.5% vs. 56.5%). Among the intervention arm participants, 80.5% had been born in Finland, while in the control arm, 88.7%. Other publications report details about recruitment,^27^ baseline associations,^32^ and findings among teachers.^26^ Data collection took place between January 11, 2015 and April 18, 2017.

### Randomization and allocation

Randomization was conducted at the school level and the matched school units (with specified educational tracks) by a statistician. Student participants were blind to randomization. Due to practical reasons,^27^ assessors were not blind to allocation but all contacts and interactions with the participants were carefully scripted in order to provide identical contacts with control and intervention participants and avoid bias. The research staff handled all communication about the study. Details of the various measures taken to reduce the risk of bias in this trial are listed in Supplementary File 1.

### Intervention

The intervention objective was to increase total PA. The intervention development aimed to ensure a good fit with the school curriculum, and teachers and student representatives were involved in the design of the intervention (for details, see^29^). For example, some activities were piloted among a small group of students and then modified based on feedback. The trial participants were not part of the co-design team. Scarce resources and minimal time available for intervention delivery in the school settings were accounted for as key constraints.

**Figure 2.**
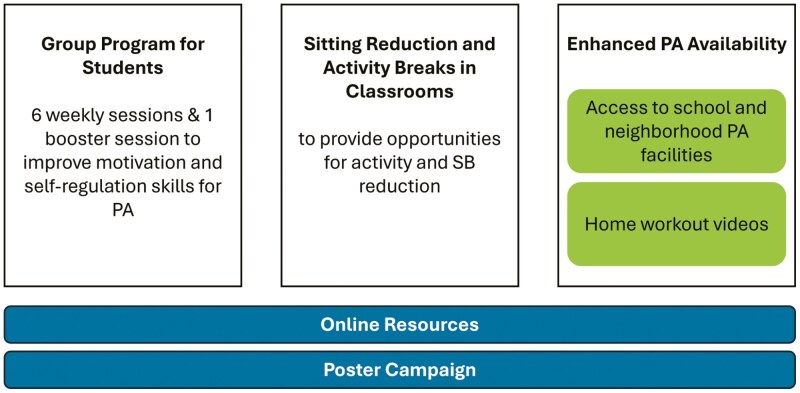
Overview of the *Let’s Move It* intervention components.

The current intervention included theoretical components that were based on the use of previous evidence, behavioral science theory, co-creation, and empirical optimization. This led to a whole school system intervention to increase PA and reduce SB. The development aimed to make the intervention acceptable, practicable, effective, cost-effective, and disseminable.^27,33^ The systematic intervention development has been described in detail,^29^ as well as the preceding comprehensive research and evidence synthesis.^26,27,29^ Activity behaviors by adolescents were identified to be affected by multiple influences.^34^ Hence, the intervention drew on several theories addressing both quantity and quality of motivation, environmental influences, and self-regulation (eg, the self-determination theory [SDT],^35^ reasoned action approach [RAA]^36^). As the teachers were identified to control a large part of students' sedentary environments during weekdays, their in-class behavior was targeted with an intervention, based on SDT, RAA, and habit theory.^37^ Systematic intervention development led to a 2-pronged approach (see Figure 2): PA behaviors were targeted through reflective motivational and self-regulation processes in face-to-face student group sessions along with graded, step-wise self-guided experimentation of self-selected PA (SDT, RAA). Sessions were delivered as part of the school curriculum, and their duration ranged from 45 to 60 min. Skills taught to the students included goal setting, planning, self-monitoring, goal review, as well as obtaining social support, and modifying their own environments. A description and manual of the sessions^29^ also detail how session activities instrumentalized theories, often targeting multiple theoretical constructs simultaneously, for example, Coping Plan Consultants exercise, where students in small groups were presented with the case of an “inactive adolescent who wants to increase their activity levels,” and then prompted the group to brainstorm ideas for this case: this exercise aimed to provide students with practical tips for exercise, build self-efficacy, provide coping planning skills, and support descriptive norms relating to exercise (see description in,^33^  Table 1). SB was addressed with environmental changes, including physical changes in classrooms (eg, gym balls as chairs) and teacher-led changes in classroom practices (eg, increasing breaks in sitting in class). Teacher training, to deliver the SB intervention, was delivered as three 2-h workshops that aimed to build teachers’ motivation, self-regulation, and habits towards using classroom practices to reduce student sitting (for a detailed description, see^26^). Both the student group sessions and the teacher's training workshops were delivered by facilitators who were part of the research team. A parallel poster campaign and increased access to other environmental opportunities for PA (eg, students were informed of the opportunity for lower-cost participation in neighborhood sports centers) boosted both approaches. Families were contacted with letters communicating key messages, and teachers and other staff were encouraged to keep up the intervention activities over the course of the study for example in their regular staff meetings.

**Table 1.** Analyses of intervention effects on primary and secondary activity outcomes.

	Intervention			Control			Between groups		
	BL mean (SD)	Change, Mean (95% CI)		BL mean (SD)	Change, Mean (95% CI)		Treatment effect, B (95% CI)		LMM*
Outcome		T3 − BL	T4 − BL		T3 − BL	T4 − BL	T3 − BL	T4 − BL	*P*-value
**Accelerometry, weekdays**									
Light PA, min/day	168.6 (51.5)	1.9 (−3.7 to 7.4)	−4.4 (−11.7 to 3.0)	169.3 (51.7)	−5.0 (−9.8 to −0.3)	−5.9 (−12.9 to 1.2)	9.3 (2.8 to 15.9)	4.6 (−4.3 to 13.4)	.01
MVPA, min/day	71.9 (31.0)	−0.7 (−4.3 to 3.0)	−6.8 (−11.1 to −2.5)	68.0 (28.3)	0.7 (−2.4 to 3.8)	−5.9 (−9.8 to −2.0)	0.2 (−4.1 to 4.5)	1.4 (−3.4 to 6.3)	.80
Steps	8658.6 (3061.0)	15.8 (−368.4 to 400)	−595.2 (−1031.9 to −158.6)	8257.6 (2915.5)	19.1 (−283.1 to 321.3)	−556.6 (−947.6 to −165.7)	199.3 (−237.4 to 636. 1)	209.9 (−276.4 to 696.3)	.73
Standing, min/day	88.5 (42.9)	−0.3 (−4.3 to 3.8)	−0.3 (−6.8 to 6.3)	86.3 (43.2)	0.6 (−3.8 to 5.0)	−1.5 (−7.1 to 4.1)	1.5 (−4.0 to 7.0)	2.5 (−4.6 to 9.6)	.95
SB	543.9 (99.5)	−32.0 (−43.2 to −20.8)	−23.8 (−37.1 to −10.4)	522.9 (110.5)	−8.6 (−19.5 to 2.3)	−5.6 (−20.7 to 9.5)	−4.3 (−15.7 to 7.2)	4.4 (−10.0 to 18.8)	.05
Breaks in SB	26.9 (7.6)	−0.4 (−1.2 to 0.5)	−1.9 (−3.0 to −0.8)	24.9 (7.5)	0.0 (−0.8 to 0.8)	−0.3 (−1.5 to 0.8)	0.8 (−0.2 to 1.8)	0.2 (−1.1 to 1.5)	.05
**Accelerometry, weekend**									
Light PA, min/day	175.2 (81.2)	−8.5 (−20.6 to 3.6)	0.6 (−14.6 to 15.8)	169.4 (77.1)	−12.1 (−22.6 to −1.6)	17.2 (3.0 to 31.5)	4.6 (−8.4 to 17.7)	−2.0 (−20.5 to 16.6)	.84
MVPA, min/day	51.2 (41.7)	0.4 (−6.0 to 6.8)	−7.3 (−14.8 to 0.3)	52.3 (42.8)	−2.3 (−8.7 to 4.0)	−1.3 (−9.5 to 7.0)	1.9 (−5.9 to 9.7)	−2.9 (−11.3 to 5.4)	.30
Steps	6380.2 (4112.4)	153.5 (−531.7 to 838.6)	−495.5 (−1275.8 to 284.9)	6530.3 (4400.5)	−222.8 (−839.5 to 394.0)	61.9 (−816.1 to 940.0)	298.2 (−493.4 to 1089.7)	−227.5 (−1125.9 to 670.8)	.24
Standing, min/day	76.7 (52.1)	−1.7 (−9.0 to 5.7)	3.9 (−6.2 to 14.1)	72.5 (45.7)	4.5 (−2.4 to 11.4)	10.1 (1.1 to 19.1)	−5.0 (−14.5 to 4.6)	1.7 (−9.7 to 13.1)	.77
SB	499.4 (146.3)	0.3 (−23.8 to 24.5)	−3.9 (−34.2 to 26.5)	500.1 (152.4)	−5.9 (−28.3 to 16.6)	10.9 (−14.2 to 35.9)	3.6 (−18.1 to 25.3)	6.9 (−22.2 to 36.1)	.79
Breaks in SB	26.3 (11.8)	−1.4 (−3.0 to 0.3)	−2.1 (−4.2 to 0.0)	24.5 (10.9)	−0.4 (−2,0 to 1.2)	1.5 (−0.4 to 3.5)	−0.5 (−2.4 to 1.4)	−0.5 (−2.9 to 1.9)	.22
**Accelerometry, school hours**									
Light PA, min/day	65.8 (22.3)	9.4 (5.9 to 12.9)	9.7 (4.8 to 14.5)	71.4 (29.3)	−3.7 (−7.5 to 0.1)	0.8 (−5.0 to 6.7)	11.2 (6.6 to 15.8)	4.6 (−2.3 to 11.5)	<.001
MVPA, min/day	35.1 (12.8)	0.2 (−1.9 to 2.3)	0.6 (−1.7 to 2.9)	31.3 (11.4)	0.1 (−1.9 to 2.0)	2.3 (−0.6 to 5.1)	2.6 (0.1 to 5.1)	2.9 (−0.3 to 6.2)	.61
Steps	4157.4 (1295.3)	183.6 (−35.1 to 402.4)	209.4 (−28.9 to 447.7)	3803.2 (1268.9)	18.2 (−196.7 to 233.0)	287.9 (−8.7 to 584.5)	388.6 (116.2 to 661.2)	335.8 (9.1 to 662.5)	.11
Standing, min/day	44.4 (25.0)	2.6 (−0.3 to 5.5)	2.2 (−2.0 to 6.5)	45.8 (27.7)	0.3 (−3.6 to 4.2)	1.2 (−3.5 to 5.8)	2.4 (−2.0 to 6.8)	1.4 (−3.8 to 6.5)	.27
SB	254.6 (40.3)	−18.2 (−24.3 to −12.0)	−22.5 (−31.0 to −14.1)	246.3 (54.3)	−4.0 (−11.8 to 3.7)	−10.9 (−21.9 to 0.1)	−13.6 (−21.9 to −5.3)	−4.5 (−15.3 to 6.2)	.001
Breaks in SB	11.6 (3.4)	1.1 (0.6 to 1.7)	0.2 (−0.4 to 0.8)	11.0 (3.6)	0.2 (−0.4 to 0.7)	0.6 (−0.3 to 1.5)	1.2 (0.5 to 1.8)	0.2 (−0.7 to 1.0)	.10
**Self-reports**									
Self-reported MVPA	2.8 (1.9)	0.26 (0.06 to 0.47)	0.11 (−0.13 to 0.34)	2.8 (1.9)	−0.03 (−0.24 to 0.18)	−0.21 (−0.43 to 0.01)	0.096 (−0.146 to 0.338)	0.119 (−0.153 to 0.391)	.13

Abbreviations: BL, Baseline; T3, Time 3, 2-month follow-up; T4, Time 4 14 month.

*Linear mixed model adjusted for gender, age, parents’ birth country, parents’ education and study track. Student group as random effect.

### Data collection procedures

The study was conducted in 6 cohorts. Data in each cohort were collected at 4-time points: pre-intervention baseline (Time 1, T1), during the intervention after the third group intervention session (T2, intervention arm only), immediately after the intensive intervention at 2 months (T3), and at 14 months (T4).^27^ The trial employed methods to enhance the quality of measurement (eg, careful, and repeated training of assessors, exchanging assessors between schools). All data were collected during school hours at schools. Students filled out the online survey, accelerometers and instructions were distributed to students, and anthropometric measures were taken.

## Measures

### Behavioral outcomes

MVPA was measured with both accelerometers and self-report, due to accelerometers missing certain types of sports (eg, swimming). To assess SB comprehensively, the primary outcomes include both the time spent sitting or lying down and breaks in sedentary time. PA was measured using accelerometers (Hookie AM 20, Traxmeet Ltd, Espoo, Finland) with a digital triaxial acceleration sensor (ADXL345; Analog Devices, Norwood MA), that has been shown to be a valid measurement tool both among adults^38^ and young people.^39^ Daily average minutes of MVPA, total sedentary time, and breaks were the co-primary accelerometer-measured outcomes. (It should be noted that due to an oversight, step count was also listed in the registration, but this paper is amended to report the 4 main outcomes as per protocol.) The accelerometers collected and stored the triaxial raw data in actual acceleration (g-units). The data were analyzed in 6 s’ epoch length. The 3 PA intensity categories based on metabolic equivalents (MET) were light, moderate, and vigorous. Light PA is defined as activity corresponding to 1.5 − 2.9 MET, moderate activity as 3.0–5.9 MET, and vigorous activity as more than 6 MET.^40^ According to the definition of SB,^41^ the time spent in sitting and reclining positions are combined to indicate SB, and time standing still is analyzed separately. Breaks in sedentary time were calculated on the basis of the number of lying/sitting periods ending up with a clear vertical acceleration. (For more information, see^27^). Participants were requested to wear the accelerometer over the right hip during waking hours, except during showers and other water activities, for 7 consecutive days. Daily reminders were texted to consenting students.^42^ Student data were included in the analysis if the accelerometer was worn for ≥ 10 h on ≥ 4 days. Weekday school hours were estimated to occur between 8 am and 3 pm, and for these analyses, the criterion was set at a minimum of 5 h during school hours.

Self-reported MVPA assessed the number of days the participant reported having been active for at least 30 min during the past week, a part of the NordPAQ measure.^43^ For further information on the MVPA measures (accelerometry and questionnaire based), see Supplementary File 2. At the time of trial design, MVPA, SB, and breaking SB had the most evidence regarding health benefits.

### Anthropometric data

Body composition, that is, lean mass and adiposity (fat mass), was measured with a Tanita (MC-780MA). Height was recorded to the nearest 0.1 cm using a portable stadiometer. Analyses controlled for height as well as season of measurement due to arm imbalance.

### Other secondary outcomes

A range of other secondary outcomes were also measured, including self-rated health, pain, and illnesses. Self-reported physical fitness was assessed with the question “My physical fitness in my opinion is currently…,” followed by 5 response options (good, rather good, satisfactory, rather poor, and poor).^44^

### Student characteristics

An online survey assessed sociodemographic characteristics, and other measures which are not included in the current paper (eg, hypothesized psychosocial mediators of intervention effect) (for a full list of measures obtained in the trial, see Table 3 in^9^).

### Process measures

Intervention fidelity—the degree to which interventions are put into practice as intended^45,46^—plays a crucial role in the reliability and validity of intervention research. However, fidelity is often overlooked in behavior change intervention studies,^46^ and it is often conceptualized only as fidelity of delivery by the intervention provider (eg, ^47^). However, assessing the extent to which participants receive the intervention, and whether this “received dose’ is associated with outcomes, is also warranted. *Delivery fidelity* was measured with checklists. These listed each intervention activity conducted during the 7 face-to-face student group sessions (eg, “I revisited the most important messages of the current session,” “I presented, prompted, and participated in the activity break,” ‘I highlighted coping planning strategies”). This checklist was answered by the session facilitator who was asked, for each class and each activity, if each intervention activity was “Delivered fully as intended,” “Partly delivered,” or “Not delivered.” *Fidelity of receipt* was here conceptualized as students' attendance in sessions, based on a record kept by facilitators. The analyses were conducted by comparing 3 groups—those not attending sessions at all, those who attended 1–3 sessions, and those who attended 4–6 of the 6 sessions.

### Statistical analyses

Longitudinal data were analyzed with Linear Mixed Models (LMM) for continuous outcome measures and Generalized Linear Mixed Models for dichotomous outcome measures. These analyses allowed the incorporation of incomplete longitudinal data into the analyses. At baseline,^32^ the 2 arms differed in the proportions of the 4 educational tracks (nurses, IT, business, and hotel and catering), and as the educational track was strongly associated with PA, the educational track was also controlled for in the analyses. Baseline values of gender, age, educational track, parental SES, and country of birth were modeled as covariates with fixed effects and student group as a covariate with a random effect. For outcomes from accelerometer data, accelerometer wear time was used as a time-varying covariate. Analyses were conducted on weekdays, weekends, and school time (8 am–3 pm).

Analyses of the change in MVPA are reported for the whole trial cohort, and for a subsample excluding those displaying high levels of MVPA at baseline. The reason for this is that the intervention components to increase PA were specifically designed for youth with low levels of PA and MVPA (ie, the programme theory does not hypothesize the effectiveness of the intervention for individuals with already high levels of PA), hypothesizing greater gains for those low in PA.^48^ For the low versus high MVPA stratification, a feasible cutoff point was set so that no more than 20% of the trial cohort would be excluded from the analysis. It should be noted that the remaining 80% may still include active individuals. The power analysis in the protocol was performed using this cutoff.^27^

The reported *P*-values are taken from interaction effects between group and time. Subgroup analyses by educational track were conducted as sensitivity analyses to evaluate the eventual differential response to intervention. Sensitivity analyses excluding over 20-year-olds and non-Finnish-speakers were also conducted.

Within-group differences between the 2 time points are presented with mean difference and 95% confidence interval. Between-group differences in changes between the 2-time points were analyzed with an analysis of covariance using the baseline value of outcome as a covariate. All analyses were performed using IBM SPSS Statistics 27.0. *T*-tests were used to determine whether students who provided data at follow-up differed from those who only provided baseline data on gender, age, and PA level. For all analyses, the significance was set at 0.05.

## Results

### Sample/enrollment

Six school units were recruited for the study, including 4 municipal and 2 private schools. Informed signed consent was received from 89.7% of the approached students. Figure 1 outlines the flow of participants. Detailed baseline characteristics of this sample have been reported elsewhere.^32^

At baseline, 81.4% of those participants who wore an accelerometer provided at least 4 days of valid accelerometer data. At 2- and 14-month follow-ups, 862 and 623 participants wore an accelerometer, and 60.4% and 65.2% of them provided at least 4 days of valid accelerometer data. Dropout was larger in the intervention arm (T3: 34.1%, T4: 26.3%) than in the control arm (T3: 24.7%, T4: 17.9%). While neither baseline age, gender, nor PA levels predicted dropout, parental education did. Participants whose parents only had basic education dropped out (27.9%) more than participants whose parents had college or equivalent degrees (11.0%) (*P* = .002).

The intervention and control arms differed in some background factors: Firstly, parental socioeconomic status was higher in the control arm (mother’s university education in the intervention arm 13.4%, among controls 20.7%; or unknown: intervention: 29.1%, control 22.7%). Secondly, there were more students born in Finland in the control arm (88%) than in the intervention arm (80%), and students whose 1 or both parents were born in Finland (control 78%, intervention 66%), reflecting the fact that the intervention arm was more ethnically diverse, much of this explained by (mostly parental) refugee backgrounds.

### Fidelity

*Fidelity of intervention delivery*, measured after each session with facilitator checklists of the session components, was high for all groups: all components were either fully or at least partly delivered 97.0 % of the time, on average. Facilitators reported not having delivered at all only 2.7% of the intended activities. The criterion of fidelity of at least 80% was fulfilled for all groups, with 8 groups receiving full 100%, and no groups receiving less than 83% of the components. The most commonly omitted components included a recap of important messages at the end of session 1 (omitted in 25.0% of the facilitator reports) and at the end of session 2 (21.4%), giving intervention letters to the students’ families (20.0%), presenting social media accounts for students at session 3 (18.2 %) or mentioning them at session 6 (13.3%), and showing the group’s PA maps at the booster session (12.0%). The most often perfectly delivered (ie, highest rating) “active ingredient” components were the feeling cards exercise at session 1 (93.3%), the discussion exercise of the *Let’s Move It* principles at session 6 (93.3.%), and problem solving at the booster session (96%) (See Supplementary File 3). *Fidelity of receipt*, measured as attendance, was adequate: Of the 6 sessions in the first 2 months, 67% of intervention arm participants attended at least 4.

### Co-primary outcomes (accelerometer-measured)

At 2 months, differences between students in the intervention and control arms were not detected in the co-primary outcomes of MVPA or SB on the whole (Table 1). There were no differences between arms in any of the activity variables (in absolute minutes) at the 14-month follow-up.

**Figure 3.**
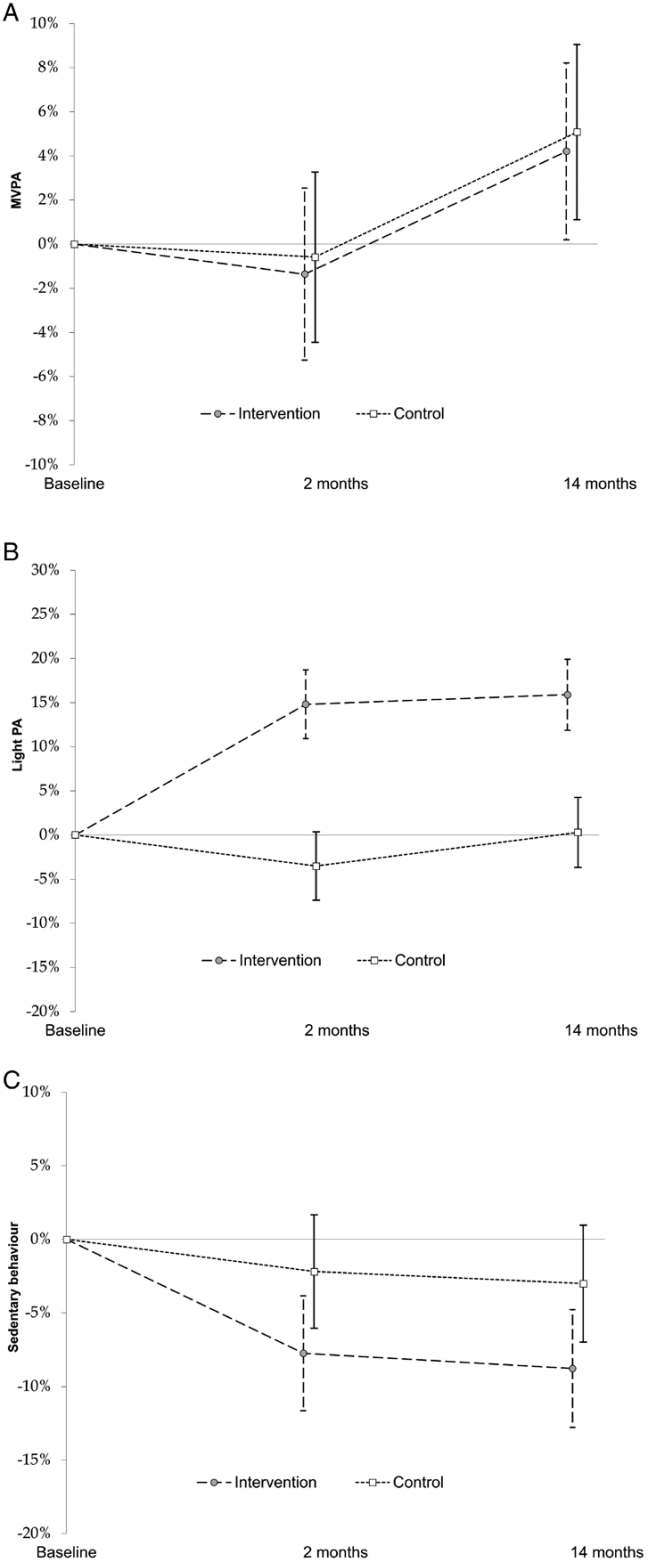
Proportional changes in moderate-to-vigorous physical activity (A), in light physical activity (secondary outcome) (B), and sedentary behavior during school time (C).

As for exploratory analyses, Table 1 shows that on weekdays, the intervention arm reduced their total daily SB time by 32 min (95% CI, −43.2 to −20.8) compared with the control arm’s reduction of only 8.6 (95% CI, −19.5 to −2.3) (*P* = .047). As for the other activity variables, the only detectable difference between arms was in light PA, with lower levels among the control arm at the 2-month follow-up (between-arm mean difference 9.3 min; 95% CI, 2.8–15.9) (*P* = .01). During school hours only (note, also exploratory analyses), changes in light PA and SB were significant (both *P* < .001), with light PA minutes significantly increasing and SB minutes significantly decreasing by the 2-month follow-up in the intervention arm compared to the control arm (between-arm difference in light PA (LPA) was 11.2 min; 95% CI 6.6–15.8 and in SB −13.6 min; 95% CI −21.9 to −5.3). The proportional differences between arms’ LPA remained at the long-term follow-up (see Figure 3C). It should be noted that the analyses differentiating school time and leisure time, as well as weekdays, and weekends were not registered, and were exploratory.

Excluding the 20% with the highest MVPA levels at baseline, there were no detectable differences in the outcome variables between arms. The educational track did not show a differential response to the intervention. Fidelity did not alter the results systematically, but, compared to those who attended no sessions, light PA improved among those who attended at least 4 of the 6 sessions (*P* = .005), whereas for those who attended 1–3 sessions, no such difference was found (*P* = .375). Also, compared to those who attended no sessions, those who attended 1–3 sessions reduced their SB more (*P* = .26), but those who attended 4–6 sessions did not differ from non-attenders (*P* = .068).

### Secondary outcomes

Between baseline and T4, the proportion of participants self-reporting poor fitness decreased in the intervention arm but increased in the control arm, but the 10.7%-point difference lost significance after controlling for confounders (Table 2). No other differences were found. No adverse events or side effects were detected.

**Table 2.** Analyses of intervention effects on secondary outcomes.

	Intervention			Control			Between groups		
Outcome	BL mean (SD)/%	Change, Mean (95% CI)		BL mean (SD)/%	Change, Mean (95% CI)		Treatment effect, B (95% CI)		LMM*
		T3 − BL	T4 − BL		T3 − BL	T4 − BL	T3 − BL	T4 − BL	*P*-value
Fat mass, kg	16.96 (9.11)	N/A	0.98 (0.60 to 1.37)	17.75 (10.13)	N/A	1.00 (0.062 to 1.37)	N/A	−0.136 (−0.765 to 0.492)	.36
Lean mass, kg	48.34 (10.00)	N/A	1.32 (1.01 to 1.62)	49.38 (10.05)	N/A	1.84 (1.59 to 2.09)	N/A	−0.601 (−1.016 to −0.185)	.68
Injuries (7-day diary)	9.2%	2.0% (−2.3% to 6.4%)	1.6% (−3.5% to 6.6.%)	10.9%	−1.1% (−5.8% to 3.4%)	−0.5% (−5.6% to 4.6%)	3.2% (1.0% to 5.4%)	2.1% (0.1% to 4.0%)	.29
Illnesses (7-day diary)	28.5%	1.9% (−4.9% to 8.8%)	3.9% (−4.5% to 12.4%)	30.20%	−4.1% (−11.2% to 3.0%)	4.9% (−3.0% to 12.7%)	6.1% (3.1% to 9.1%)	−0.9% (−4.8% to 2.9%)	.55
Injuries (questionnaire)	6.9%	0.8% (−1.7% to 3.4%)	1.6% (−2.2% to 5.5%)	8.9%	−0.7% (−3.8% to 2.3%)	0.0% (−3.5% to 3.5%)	1.6% (0.4% to 2.7%)	1.6% (0.2% to 3.1%)	.65
Illnesses (questionnaire)	6.5%	2.1% (−0.9% to 5.2%)	−1.0% (−4.6% to 2.6%)	6.4%	1.4% (−1.9% to 4.8%)	3.6% (−0.2% to 7.4%)	0.7% (−1.0% to 2.4%)	−4.6% (−6.8% to −2.4%)	.31
Neck and shoulder pain symptoms	56.2%	2.7%(−1.8% to 7.2%)	5.2% (−0.7% to 11.0%)	56.5%	2.2% (−2.2% to 6.6%)	4.1% (−1.2% to 9.4%)	0.5% (−1.6% to 2.6%)	1.1% (−2.2% to 4.4%)	.63
Lower back pain symptoms	49.9%	4.5% (−0.1% to 9.0%)	3.7% (−2.3% to 9.8%)	50.0%	−0.5% (2.9% to 7.0%)	0.3% (−5.5% to 6.1%)	5.0% (2.9% to 7.0%)	3.5% (1.2% to 5.7%)	.59
Headache symptoms	62.1%	3.6% (−0.7% to 7.9%)	2.8% (−3.0% to 8.5%)	59.8%	7.2% (2.8% to 11.6%)	7.1% (2.0% to 12.2%)	−3.6% (−6.7% to −0.6%)	−4.3% (−7.6% to −1.0%)	.73
Self-reported fitness, poor	47.5%	−0.6% (−4.9% to 3.6%)	−2.6% (−7.9% to 2.6%)	43.9%	2.1% (−2.5% to 3.6%)	8.1% (2.4% to 13.8.%)	−2.8% (−4.3% to −1.2%)	−10.7% (−14.1% to 7.3%)	.17

Abbreviations: BL, Baseline; T3, Time 3, 2-month follow-up; T4, Time 4 14 month.

Pain symptom frequencies ranging from once a month to almost daily.

Linear mixed models adjusted for gender, age, parents’ birth country, parents’ education, and study track. Student group as random effect. For far mass and lean mass, the linear mixed models were also adjusted for the time of the year.

Figure 3 visually displays the proportional changes in MVPA, SB (primary outcomes), and LPA (secondary outcome) in both arms. Supplementary Tables display analyses without weekday-weekend separation (in accordance with the study statistical protocol registration), and without adjustments for confounding variables.

### Gender differences

Overall, the intervention seemed to be more effective among female than male participants (see Supplementary File). Exploratory analyses indicated that during weekdays, the intervention effect on sedentary time was significant among females (*P* = .002) but not among males (*P = *.492), as well as on LPA (females *P = *.003 vs. males *P = *.764), MVPA (females *P = *.045 vs. males *P = *.091) and self-reported MVPA (females *P = *.047 vs. males *P* = .825). The effect on breaks in SB was non-significant among both genders (females *P* = .261, males *P = *.056).

## Discussion

This cluster-randomized controlled trial (RCT) investigated the effects of a multi-component intervention delivered in vocational schools on student activity behaviors and secondary outcomes. The trial did not find evidence that the intervention was effective in changing the co-primary outcomes of objectively measured MVPA or SB overall. However, there was a statistically significant increase in LPA during weekdays, and a decrease in SB during school hours, with on average, 9 min more LPA during weekdays, and 11 min more LPA, and 14 min less SB during school hours. Gender-specific analyses suggested the intervention was more effective among females. We highlight that the analyses regarding school, weekday and weekend, or analyses by gender, were not pre-registered, but were conducted to achieve a better understanding of the trial.

The results align with those from other trials: Most school-based interventions have not produced meaningful improvements in accelerometer-measured MVPA.^19,23^ The qualitative process evaluation suggested that intervention arm students identified several insights regarding MVPA, along with useful skills and ideas, saying these also inspired them to increase activity.^49^ Still, many of them reported not taking up PA in their daily lives.^50^ Indeed, the current analyses also suggest that these insights or improved motivation were not sufficiently widespread to translate into measurable behavior outside school hours, especially among males.

However, an increased volume of light PA and decreased SB during school time were observed, indicating that some SB time had been replaced by light PA. This is likely attributable to successful teacher activation of classroom time,^26^ and also reflected in positive, yet short-lived, improvements in SB-related social cognitions among intervention-arm students.^25^ The intervention content may have been more prone to increase light PA (rather than MVPA), for example, with the messages emphasizing “starting small” and “any activity is better than nothing.” For some analyses, the change in SB is not significant given its high levels to start with in comparison to the total minutes of light PA or MVPA. Besides, increased light PA during school hours may have influenced students’ perceived need to increase PA in general and MVPA in particular, in other settings. However, this compensation hypothesis is not supported by data. The light PA finding is among the most robust in this study. It is a positive signal, as LPA has recently been found to produce health benefits,^12^ and the average weekly addition of about 50 min of LPA during the weekdays may produce significant health outcomes if sustained.

The exploratory analyses suggesting that the intervention was more effective among females, in increasing MVPA and light PA and reducing SB time, are in line with previous evidence on gender differences,^16^ although meta-analysis on trials using accelerometry found no effectiveness disparity by gender.^19^ For PA, gender-targeted interventions may be slightly more effective than gender neutral.^51^ The *Let’s Move It* intervention contained some components potentially more relevant to girls, for example, offering a critical lens on social pressure for good looks versus well-being as the primary motive for PA. Also, although male students were equally engaged as females in our co-design and feasibility study, and the art directors involved in designing the visuals and slogans of the intervention were men, our development and provider team consisted mostly of women. It is uncertain whether the decisions undertaken by a team of men would have led to an intervention more appealing to male students. It should be noted that gender is confounded with the educational track.^32^

Fidelity for some components of the intervention was good and, for others, lower than ideal. A criterion of at least 80% delivery of program components was mainly followed for all groups. However, facilitator self-reports may be biased, and therefore also a subjective report of the delivery of the intervention compromises the reliability of “presence at sessions” as an indicator of fidelity of receipt among participants. For MVPA, the programme theory hypothesized the relevance of (1) getting motivated, (2) adopting self-regulatory skills, and (3) behavioral experiments (students were encouraged to select and plan some leisure-time PA activity). The fidelity of receipt, by students, of motivational and self-regulation sessions was deemed good and adequate (see qualitative analysis of receipt^50^), but engagement with behavioral experiments and home workout videos was extremely low. The original rationale was that regularly incorporating PA in daily life—as opposed to the school curriculum only—may be more likely to result in maintenance over time, even outside of school. A better solution might have been to embed simultaneously a Physical Education class where engagement with the target behavior could have been done with high fidelity (see eg,^52^), to strengthen the effect of the motivational, self-regulatory discussion classes. However, embedding high-intensity interval training (HIIT) sessions in school hours has not resulted in sustained activity improvements.^53^ A combination of both high-quality motivational content over the school year, positive experiences during school, as well as support in identifying leisure-time PA opportunities may prove a promising combination.

Indeed, more intensive and longer interventions have been shown to be more effective^54^ as behavior change is difficult and hardly easy to learn even for the motivated ones within a few contact hours, hence, only 6 sessions targeting motivation and self-regulation may represent a too small dose to change MVPA. Our other evaluation studies^24–26,49,50^ indicate that many of the *Let’s Move It* elements were supportive of change, but the dose was too small—with the exception of the classroom-related changes, which increased LPA. What were the potentially missed opportunities that might have improved effectiveness? Recent evidence highlights the benefits of comprehensive whole-school^23^ and multi-component^22^ approaches. For MVPA, daily living arrangements and environments at home and with friends may be more influential than the school environment, and thus, activities engaging families, neighborhoods, and friends may have been called for. Although this intervention encouraged social support in several ways, an even more social network-underpinned approach, via, for example, collaborative planning with friends (see also, eg,^55^), may have been more effective, yet more difficult to deliver. With increasing appealing digital—and physically passive—screen activities, PA interventions may have to take into account such competing forces: even a strong motivation for PA may not overpower motivations for alternative fun activities such as gaming and social media.^56^ Passive overall life environments, conducive to sustaining previous passive behaviors, can indeed act as deep “valleys” in the behavioral landscape, making change particularly challenging.^57^ Furthermore, this intervention aimed to balance between offering sufficiently prepared, behavioral-science-based materials and activities (to reduce the burden on staff), yet allowing for school-specific tailoring. However, a more intensive context-specific approach may be called for.^58^ Sensitivity checks, made by removing adults from our sample, revealed similar patterns of results, which suggests that student age was not associated with intervention effectiveness.

This study has several strengths: It overcomes several methodological weaknesses of previous trials of school-based interventions in this age group,^18^ with its larger sample, using various ways to ensure and assess fidelity, and assessing outcomes with accelerometers rather than self-report only. Furthermore, the intervention itself was a result of a careful development process involving stakeholders and co-creation, behavioral theories, and its content is comprehensively reported. Finally, the trial design with mixed-methods data collection enables complementing this main outcome evaluation by process evaluation (eg,^49,50^). To the best of our knowledge, this is the first major trial of this kind in vocational schools (see also^17^).

The study has also its limitations. First, although the recruitment rate was high, suggesting low selection bias, dropout leaves results open for bias. We had originally estimated needing a post-dropout sample of 704, but some of the analyses examining change include less than 400 observations, as we followed internationally accepted procedures by excluding participants with insufficient wear time. Overall, retention rates were relatively high for this population. Also, PA levels may be different if the measurement week was not during normal school time (indeed at the final follow-up, many were on a remote learning period or an internship/training at work). Fourth, although accelerometer data give in general less subjective results than self-reports^59^ and were considered a golden standard at the time of trial design, it is somewhat restricted in type of PA. The accelerometer, as a technical device that needs to be worn for measurement to occur, suffers from reliability and validity issues, such as selective non-wear, restricted battery life, malfunction, and non-adherence. Due to restrictions on accelerometer use at work and certain sports (ie, water or contact sports), there is a risk of missing valuable information on the duration and intensity of PA. Also, accelerometer reporting of PA in intervention studies in general has been poor,^60^ hindering comparison to other similar studies. However, the strengths of accelerometry include precise data on intensity and total volume of PA during short time periods, characteristics inaccessible with self-report: For example, the number of breaks, light PA minutes, and steps could not have been assessed and researched without accelerometers.

Finally, the arms differed at baseline, with the control arm being slightly more affluent and displaying slightly more positive patterns of PA-relevant variables (see also^32^). It is difficult to assess how this might have affected the outcomes. Balancing control and intervention arms across a variety of measures in a vocational school with different educational tracks with inherent gender imbalance is challenging, if not impossible. In vocational schools, where student groups are formed based on educational tracks and the consistency of schools (as to educational tracks) varies, it is challenging to create classical cluster-RCTs, compared to, for example, high schools. Hence, the unique histories of the schools, or their students could have influenced the results. However, we were unable to analyze this due to the small number (6) of participating schools and the lack of information on pre-baseline trends. These results are in line with recent calls for systems-level interventions to target multiple socio-ecological levels, and clearly highlight the concerns on the limitations of a classical RCT design.^61^ Most similar trials have had a high risk of bias.^23^ It should also be noted that changes in external disturbances (eg, co-occurring governmental cuts in school budgets, and major changes in intake criteria, including no more requiring language proficiency among new students) may have worsened the odds for success.

## Conclusions

This was the first large trial to investigate a multi-level, multi-component school-based intervention on PA and SB in the vocational school setting. The findings suggest that leisure-time PA or SB may be too challenging, particularly among males, to change by a relatively low-intensive school-based intervention, that is, with a maximum of 7 teaching hours with groups. This aligns with the findings of school-based trials in also younger age groups.^19^ Despite positive outcomes on school-time SB and light PA, more comprehensive environmental changes may be needed to meaningfully improve total activity. Indeed, while school-based PA interventions have not managed to influence accelerometer-measured PA,^19^ this intervention seems to have caused sustainable changes also in females’ accelerometer-measured activity, which may be remarkable. Further studies should examine this gender difference. Recommendations for future research include further high-quality intervention research (see also^3^), with rigorous methodology and transparent reporting of intervention development processes^62^ and intervention content and fidelity, using not only the outcome but also process evaluations.

## Supplementary Material

kaaf023_suppl_Supplementary_Files_1

kaaf023_suppl_Supplementary_Files_2

kaaf023_suppl_Supplementary_Files_3

kaaf023_suppl_Supplementary_Files_4

## Data Availability

De-identified data from this study are available in a protected archive, Finnish Social Science Data Archive (https://www.fsd.tuni.fi/en/). Data can be obtained for research purposes by contacting the customer service at user-services.fsd@tuni.fi.

## References

[1] Strain T, Flaxman S, Guthold R, et al; Country Data Author Group. National, regional, and global trends in insufficient physical activity among adults from 2000 to 2022: a pooled analysis of 507 population-based surveys with 5·7 million participants. Lancet Glob Health. 2024;12:e1232-e1243. https://doi.org/10.1016/S2214-109X(24)00150-5 38942042 PMC11254784

[2] Kämppi K, Aira A, Halme N, et al Results from Finland’s 2018 Report Card on physical activity for children and youth. J Phys Act Health. 2018;15:S355-S356. https://doi.org/10.1123/jpah.2018-0510 30475141

[3] van Sluijs EMF, Ekelund U, Crochemore-Silva I, et al Physical activity behaviours in adolescence: current evidence and opportunities for intervention. Lancet (London, England). 2021;398:429-442. https://doi.org/10.1016/S0140-6736(21)01259-9 34302767 PMC7612669

[4] Husu P, Tokola K, Vähä-Ypyä H, et al Physical activity has decreased in Finnish children and adolescents from 2016 to 2022. BMC Public Health. 2024;24:1343. https://doi.org/10.1186/s12889-024-18854-7 38762462 PMC11102264

[5] Elgar FJ, Pförtner T-K, Moor I, De Clercq B, Stevens GWJM, Currie C. Socioeconomic inequalities in adolescent health 2002–2010: a time-series analysis of 34 countries participating in the Health Behaviour in School-aged Children study. Lancet. 2015;385:2088-2095. https://doi.org/10.1016/s0140-6736(14)61460-4 25659283

[6] Beenackers MA, Kamphuis CB, Giskes K, et al Socioeconomic inequalities in occupational, leisure-time, and transport related physical activity among European adults: a systematic review. Int J Behav Nutr Phys Act. 2012;9:116. https://doi.org/10.1186/1479-5868-9-116 22992350 PMC3491027

[7] SFM C, Van Cauwenberg J, Maenhout L, Cardon G, Lambert EV, Van Dyck D. Inequality in physical activity, global trends by income inequality and gender in adults. Int J Behav Nutr Phys Act. 2020;17:142. https://doi.org/10.1186/s12966-020-01039-x 33239036 PMC7690175

[8] The Lancet Public Health. Time to tackle the physical activity gender gap. Lancet Public Health. 2019;4:e360. 31345750 10.1016/S2468-2667(19)30135-5

[9] World Health Organization. WHO guidelines on physical activity and sedentary behaviour [Internet]. World Health Organization; 2020. Accessed April 4, 2020. https://www.who.int/publications-detail-redirect/9789240015128

[10] 2018 Physical Activity Guidelines Advisory Committee. 2018 Physical Activity Guidelines Advisory Committee Scientific Report. Washington, DC: U.S. Department of Health and Human Services, 2018. Accessed April 4, 2020. https://health.gov/sites/default/files/2019-09/PAG_Advisory_Committee_Report.pdf

[11] Piercy KL, Troiano RP, Ballard RM, et al The physical activity guidelines for Americans. JAMA. 2018;320:2020-2028. https://doi.org/10.1001/jama.2018.14854 30418471 PMC9582631

[12] Chastin SFM, Craemer MD, Cocker KD, et al How does light-intensity physical activity associate with adult cardiometabolic health and mortality? Systematic review with meta-analysis of experimental and observational studies. Br J Sports Med. 2019;53:370-376. 29695511 10.1136/bjsports-2017-097563 PMC6579499

[13] Bar-Or O. Juvenile obesity: is school-based enhanced physical activity relevant? Arch Pediatr Adolesc Med. 2005;159:996-997. https://doi.org/10.1001/archpedi.159.10.996 16203950

[14] Jones M, Defever E, Letsinger A, Steele J, Mackintosh KA. A mixed-studies systematic review and meta-analysis of school-based interventions to promote physical activity and/or reduce sedentary time in children. J Sport Health Sci. 2020;9:3-17. https://doi.org/10.1016/j.jshs.2019.06.009 31921476 PMC6943767

[15] Neil-Sztramko SE, Caldwell H, Dobbins M. School-based physical activity programs for promoting physical activity and fitness in children and adolescents aged 6 to 18. Cochrane Database Syst Rev. 2021;9:CD007651. https://doi.org/10.1002/14651858.CD007651.pub3 34555181 PMC8459921

[16] Yildirim M, van Stralen MM, Chinapaw MJM, et al; Energy-Consortium. For whom and under what circumstances do school-based energy balance behavior interventions work? Systematic review on moderators. Int J Pediatric Obes. 2011;6:e46-e57. https://doi.org/10.3109/17477166.2011.566440 PMC3190836 21651421

[17] Grüne E, Popp J, Carl J, Pfeifer K. What do we know about physical activity interventions in vocational education and training? A systematic review. BMC Public Health. 2020;20:978. https://doi.org/10.1186/s12889-020-09093-7 32571295 PMC7309979

[18] Hynynen S-T, van Stralen MM, Sniehotta FF, et al A systematic review of school-based interventions targeting physical activity and sedentary behaviour among older adolescents. Int Rev Sport Exerc Psychol. 2016;9:22-44. https://doi.org/10.1080/1750984X.2015.1081706 26807143 PMC4706019

[19] Love R, Adams J, van Sluijs EMF. Are school-based physical activity interventions effective and equitable? A meta-analysis of cluster randomized controlled trials with accelerometer-assessed activity. Obes Rev [Internet]. 2019;20:859-870. https://doi.org/10.1111/obr.12823 30628172 PMC6563481

[20] van Sluijs EMF, McMinn AM, Griffin SJ. Effectiveness of interventions to promote physical activity in children and adolescents: systematic review of controlled trials. BMJ. 2007;335:703. https://doi.org/10.1136/bmj.39320.843947.BE 17884863 PMC2001088

[21] Naylor P-J, McKay HA. Prevention in the first place: schools a setting for action on physical inactivity. Br J Sports Med. 2009;43:10-13. https://doi.org/10.1136/bjsm.2008.053447 18971250

[22] Pulling Kuhn A, Stoepker P, Dauenhauer B, Carson RL. A systematic review of multi-component Comprehensive School Physical Activity Program (CSPAP) interventions. Am J Health Promot. 2021;35:1129-1149. https://doi.org/10.1177/08901171211013281 33955278

[23] Contardo Ayala AM, Parker K, Mazzoli E, et al Effectiveness of intervention strategies to increase adolescents’ physical activity and reduce sedentary time in secondary school settings, including factors related to implementation: a systematic review and meta-analysis. Sports Med - Open. 2024;10:25. https://doi.org/10.1186/s40798-024-00688-7 38472550 PMC10933250

[24] Palsola M, Araújo-Soares V, Hardeman W, et al Evaluating the Let’s Move It intervention programme theory for adolescents’ physical activity: theorised psychosocial mechanisms of behavioural changes. Br J Health Psychol 2025; 30:e12744. https://doi.org/10.1111/bjhp.12744 39317658 PMC11586702

[25] Aulbach MB, Puukko S, Palsola M, et al How does a school-based intervention impact students’ social cognitions on reducing sedentary behavior over 14 months? Psychol Health Med. 2023;29:1235-1249. https://doi.org/10.1080/13548506.2023.2285734 38013166

[26] Köykkä K, Absetz P, Araújo-Soares V, Knittle K, Sniehotta FF, Hankonen N. Combining the reasoned action approach and habit formation to reduce sitting time in classrooms: outcome and process evaluation of the Let’s Move It teacher intervention. J Exp Soc Psychol. 2019;81:27-38. https://doi.org/10.1016/j.jesp.2018.08.004

[27] Hankonen N, Heino MTJ, Araújo-Soares V, et al “Let’s Move It”—a school-based multilevel intervention to increase physical activity and reduce sedentary behaviour among older adolescents in vocational secondary schools: a study protocol for a cluster-randomised trial. BMC Public Health. 2016;16:451. https://doi.org/10.1186/s12889-016-3094-x 27229682 PMC4882860

[28] Li N, Zhao P, Diao C, et al; ISCOLE Research Group. Joint associations between weekday and weekend physical activity or sedentary time and childhood obesity. Int J Obes (Lond). 2019;43:691-700. https://doi.org/10.1038/s41366-019-0329-9 30705394 PMC6445763

[29] Hankonen N, Absetz P, Araújo-Soares V. Changing activity behaviours in vocational school students: the stepwise development and optimised content of the “let’s move it” intervention. Health Psychol Behav Med. 2020;8:440-460. https://doi.org/10.1080/21642850.2020.1813036 34040880 PMC8114352

[30] Eldridge S, Kerry S. A Practical Guide to Cluster Randomised Trials in Health Services Research. John Wiley & Sons; 2012.

[31] Heino M. Power calculation spreadsheet for cluster randomised trials. 2019. Accessed December 11, 2019. https://osf.io/vrjp3/

[32] Heino M, Knittle K, Fried E, et al Visualisation and network analysis of physical activity and its determinants: Demonstrating opportunities in analysing baseline associations in the Let’s Move It trial. Health Psychol Behav Med. 2019;7:269-289. 34040851 10.1080/21642850.2019.1646136 PMC8114395

[33] Hankonen N, Heino MTJ, Hynynen S-T, et al Randomised controlled feasibility study of a school-based multi-level intervention to increase physical activity and decrease sedentary behaviour among vocational school students. Int J Behav Nutr Phys Act. 2017;14:37. https://doi.org/10.1186/s12966-017-0484-0 28327174 PMC5361824

[34] Hankonen N, Heino MTJ, Kujala E, et al What explains the socioeconomic status gap in activity? Educational differences in determinants of physical activity and screen time. BMC Public Health. 2017;17:144. https://doi.org/10.1186/s12889-016-3880-5 28143461 PMC5286840

[35] Ryan RM, Deci EL. Self-determination theory and the facilitation of intrinsic motivation, social development, and well-being. Am Psychol. 2000;55:68-78. https://doi.org/10.1037//0003-066x.55.1.68 11392867

[36] Fishbein M, Ajzen I. Predicting and Changing Behavior: The Reasoned Action Approach. Psychology Press; 2011.

[37] Lally P, Gardner B. Promoting habit formation. Health Psychol Rev. 2011;7:S137-S158. https://doi.org/10.1080/17437199.2011.603640

[38] Vähä-Ypyä H, Vasankari T, Husu P, Suni J, Sievänen H. A universal, accurate intensity-based classification of different physical activities using raw data of accelerometer. Clin Physiol Funct Imaging. 2015;35:64-70. https://doi.org/10.1111/cpf.12127 24393233

[39] Aittasalo M, Vähä-Ypyä H, Vasankari T, Husu P, Jussila A-M, Sievänen H. Mean amplitude deviation calculated from raw acceleration data: a novel method for classifying the intensity of adolescents’ physical activity irrespective of accelerometer brand. BMC Sports Sci Med Rehabil. 2015;7:18. https://doi.org/10.1186/s13102-015-0010-0 26251724 PMC4527117

[40] Vähä-Ypyä H, Vasankari T, Husu P, et al Validation of cut-points for evaluating the intensity of physical activity with accelerometry-based Mean Amplitude Deviation (MAD). PLoS One. 2015;10:e0134813. https://doi.org/10.1371/journal.pone.0134813 26292225 PMC4546343

[41] Sedentary Behaviour Research Network. Letter to the Editor: Standardized use of the terms “sedentary” and “sedentary behaviours.” Appl Physiol Nutr Metab. 2012;37:540-542. 22540258 10.1139/h2012-024

[42] Heino M, Knittle K, Haukkala A, Vasankari T, Hankonen N. Simple and rationale-providing SMS reminders to promote accelerometer use: a within-trial randomised trial comparing persuasive messages. BMC Public Health. 2018;18:1352. 30526616 10.1186/s12889-018-6121-2 PMC6286544

[43] Fagt S, Andersen LF, Anderssen SA, et al Nordic Monitoring on Diet, Physical Activity and Overweight: Validation of Indicators [Internet]. Nordic Council of Ministers; 2011 Accessed December 11, 2019. https://orbit.dtu.dk/en/publications/nordic-monitoring-on-diet-physical-activity-and-overweight-valida

[44] National Public Health Institute. Health 2000 Survey: Methodology Report. National Public Health Institute; 2008.

[45] Carroll C, Patterson M, Wood S, Booth A, Rick J, Balain S. A conceptual framework for implementation fidelity. Implement Sci. 2007;2:40. https://doi.org/10.1186/1748-5908-2-40 18053122 PMC2213686

[46] Toomey E, Hardeman W, Hankonen N, et al Focusing on fidelity: narrative review and recommendations for improving intervention fidelity within trials of health behaviour change interventions. Health Psychol Behav Med. 2020;8:132-151. https://doi.org/10.1080/21642850.2020.1738935 34040865 PMC8114368

[47] Hankonen N. Participants’ enactment of behavior change techniques: a call for increased focus on what people do to manage their motivation and behavior. Health Psychol Rev. 2021;15:185-194. https://doi.org/10.1080/17437199.2020.1814836 32967583

[48] Moore SC, Patel AV, Matthews CE, et al Leisure time physical activity of moderate to vigorous intensity and mortality: a large pooled cohort analysis. PLoS Med [Internet]. 2012;9:e1001335. https://doi.org/10.1371/journal.pmed.1001335 23139642 PMC3491006

[49] Kostamo K, Jallinoja P, Vesala KM, Araújo-Soares V, Sniehotta FF, Hankonen N. Using the critical incident technique for qualitative process evaluation of interventions: the example of the “Let’s Move It” trial. Soc Sci Med (1982). 2019;232:389-397. https://doi.org/10.1016/j.socscimed.2019.05.014 31146148

[50] Palsola M, Renko E, Kostamo K, Lorencatto F, Hankonen N. Thematic analysis of acceptability and fidelity of engagement for behaviour change interventions: the Let’s Move It intervention interview study. Br J Health Psychol [Internet]. 2020;25:772-789. https://doi.org/10.1111/bjhp.12433 32568447

[51] Sharkey T, Whatnall MC, Hutchesson MJ, et al Effectiveness of gender-targeted versus gender-neutral interventions aimed at improving dietary intake, physical activity and/or overweight/obesity in young adults (aged 17–35 years): a systematic review and meta-analysis. Nutr J. 2020;19:78. https://doi.org/10.1186/s12937-020-00594-0 32731865 PMC7393713

[52] Sutherland R, Campbell E, Lubans DR, et al “Physical Activity 4 Everyone” school-based intervention to prevent decline in adolescent physical activity levels: 12 month (mid-intervention) report on a cluster randomised trial. Br J Sports Med. 2016;50:488-495. https://doi.org/10.1136/bjsports-2014-094523 26359346 PMC4853531

[53] Lubans DR, Smith JJ, Eather N, et al Time-efficient intervention to improve older adolescents’ cardiorespiratory fitness: findings from the “Burn 2 Learn” cluster randomised controlled trial. Br J Sports Med. 2021;55:751-758. https://doi.org/10.1136/bjsports-2020-103277 PMC8223670 33355155

[54] Oldenburg B, Absetz P, Chan CKY. Behavioral interventions for prevention and management of chronic disease. In: Steptoe A, ed. Handbook of Behavioral Medicine: Methods and Applications [Internet]. Springer New York; 2010:969-988. https://doi.org/10.1007/978-0-387-09488-5_62

[55] Renko E, Kostamo K, Hankonen N. Uptake of planning as a self‐regulation strategy: adolescents’ reasons for (not) planning physical activity in an intervention trial. Br J Health Psychol. 2022;27:1209-1225. https://doi.org/10.1111/bjhp.12595 35451544 PMC9790213

[56] Khan A, Lee E-Y, Rosenbaum S, Khan SR, Tremblay MS. Dose-dependent and joint associations between screen time, physical activity, and mental wellbeing in adolescents: an international observational study. Lancet Child Adolesc Health. 2021;5:729-738. https://doi.org/10.1016/S2352-4642(21)00200-5 34384543

[57] Heino M, Proverbio D, Marchand G, Resnicow K, Hankonen N. Attractor landscapes: a unifying conceptual model for understanding behaviour change across scales of observation. Health Psychol Rev. 2022;0:1-18. 10.1080/17437199.2022.2146598 PMC10261543 36420691

[58] Jago R, Salway R, House D, et al Rethinking children’s physical activity interventions at school: a new context-specific approach. Front Public Health [Internet]. 2023;11. Accessed October 25, 2023. https://www.frontiersin.org/articles/10.3389/fpubh.2023.1149883 10.3389/fpubh.2023.1149883 PMC10133698 37124783

[59] Matthews CE, Hagströmer M, Pober DM, Bowles HR. Best practices for using physical activity monitors in population-based research. Med Sci Sports Exerc. 2012;44:S68-S76. https://doi.org/10.1249/MSS.0b013e3182399e5b 22157777 PMC3543867

[60] Montoye AHK, Moore RW, Bowles HR, Korycinski R, Pfeiffer KA. Reporting accelerometer methods in physical activity intervention studies: a systematic review and recommendations for authors. Br J Sports Med. 2018;52:1507-1516. https://doi.org/10.1136/bjsports-2015-095947 27539504

[61] Deaton A, Cartwright N. Reflections on randomized control trials. Soc Sci Med (1982). 2018;210:86-90. https://doi.org/10.1016/j.socscimed.2018.04.046 29731150

[62] Araújo-Soares V, Hankonen N, Presseau J, Rodrigues A, Sniehotta FF. Developing behavior change interventions for self-management in chronic illness: an integrative overview. Eur Psychol. 2018;24:7-25. https://doi.org/10.1027/1016-9040/a000330 31496632 PMC6727632

