# Supplementary material for: Effectiveness of the Let’s Move It multi-level vocational school-based intervention on physical activity and sedentary behavior: a cluster randomized trial
Source: Ann Behav Med. 2025 May 27;59(1):kaaf023. doi: 10.1093/abm/kaaf023 (PMC12169330; doi:10.1093/abm/kaaf023)
Supplement: kaaf023_suppl_Supplementary_Files_1 [file kaaf023_suppl_supplementary_files_1.docx]

**Supplementary file 1.** Risk of Bias Justification (RATIONALE) table (de Bruijn et al., 2015) for reducing risk of bias in health behavior change interventions as applied to the Let’s Move It cluster randomized trial.

| **Type of bias** | **Common strategies for reducing risk of bias** | **Common strategies applied in trial?**  **If yes: How was it implemented?**  **If no: Why not?** |
| --- | --- | --- |
| SELECTION BIAS | Random sequence generation | **Yes**: Computer generated pseudo random numbers (Mersenne-Twister algorithm) were used in randomization. |
|  | Concealment of allocation until assigned | **Yes**: Participants in both arms were not informed which arm they have been randomized into before completion of baseline measurements. |
| RECRUITMENT BIAS | Include participants before randomization (or)  blind recruiters for cluster assignment | **Yes**: School clusters were included and only then randomized.  **No**: Not possible for practical reasons. |
| BASELINE IMBALANCE (chance bias) | Include sufficiently large sample size | **Yes**: At baseline, target N was 1400 students. |
|  | Control for key prognostic covariates | **Yes**: Clusters were matched based on the educational track (correlate of PA). |
| PERFORMANCE BIAS | Blind personnel for treatment assignment  (or)  use a strict protocol for participant contact | **No**: Not possible for practical reasons.  **Yes**: Used a strict protocol for participant contact. |
| DETECTION BIAS | Objective outcome measure  (or)  blinding outcome assessors to group assignment | **Yes**: Accelerometry and bioimpedance measurement. Additionally, research staff collecting data received continuous training to ensure all contact between staff and participants was similar across both arms, in e.g. in advising the use of accelerometers.  **No**: Not possible for practical reasons. |
| ATTRITION BIAS | Analyze participants as randomized | **Yes**: Intention to treat analyses applied. |
|  | Advanced data imputation procedures | **No**: For the current reported analyses multiple imputation was not used as, by protocol, the main results will be analysed and published as observed. |
| REPORTING BIAS | Online registration trial protocol | **Yes**: Trial was registered in ISRCTN registry. |
|  | CONSORT guideline reporting | **Yes**: CONSORT guideline was followed in reporting. |
| CONTAMINATION BIAS | Blinding for  treatment  assignment [or]  Cluster  randomization [or]  Restrict access to  intervention  materials | **No**: Not possible as control arm treatment was standard curriculum.  **Yes**: Cluster randomization (school level).    **Yes**: Access to intervention materials was restricted, i.e., intervention teachers and students were instructed to keep the posters, workbooks and slides etc.to themselves. Online materials were password protected. |
|  | Control for  contamination in the  analyses | **No**: Due to safeguarding of the intervention materials (see above), contamination is very unlikely to have happened. |
| Inappropriate  administration | Promote accurate  program delivery | **Yes**: Student and teacher intervention facilitators followed a clear manual. Facilitators were continuously trained (incl. simulations). |
|  | Control for variable  program delivery | **Yes**: Promoting and monitoring of fidelity of delivery using facilitator checklists for both session component delivery and interaction style in delivery. Written plan for scheduling of the poster campaign. |
| Stop early  /continue for  benefit | Report sample size  computation in the  study protocol | **Yes**: Was reported in protocol. |
|  | Report planned  interim analyses in  the study protocol  and apply  appropriate analyses | **Yes**: Interim analyses, as specified under Statistical analyses in the protocol, were conducted on the internal pilot data (including outcomes and theoretical mediators) in order to obtain intra-class correlation estimates to inform power calculations, as well as to detect unintended consequences. |
| Scientific  misconduct | Be transparent about  study methods  before, during and  after trial | **Yes**: Methods were registered before completing the baseline measurements in the ISRCTN Registry. |
|  | Monitoring conduct  by team of study  board | **Yes**. Regular consultation in problematic situations with the steering group. |
|  | Minimize effects of  vested interest | **Yes**. Entire team committing to not engaging in scientific misconduct and agreeing that in the best interest of all is to use proper scientific methods and minimize bias. Sharing of data in collaboration with the Finnish Social Science Data Archive FSD. |

Reference: de Bruin, M., McCambridge, J. & Prins, J. M. (2015) Reducing the risk of bias in health behaviour change trials: Improving trial design, reporting or bias assessment criteria A review and case study, *Psychology & Health*, 30:1, 8-34, DOI: [10.1080/08870446.2014.953531](https://doi.org/10.1080/08870446.2014.953531)
