# Supplementary material for: Effectiveness of the Let’s Move It multi-level vocational school-based intervention on physical activity and sedentary behavior: a cluster randomized trial
Source: Ann Behav Med. 2025 May 27;59(1):kaaf023. doi: 10.1093/abm/kaaf023 (PMC12169330; doi:10.1093/abm/kaaf023)
Supplement: kaaf023_suppl_Supplementary_Files_2 [file kaaf023_suppl_supplementary_files_2.docx]

**Supplementary file 2: Details about considerations for MVPA assessment methods**(Effectiveness of the Let’s Move It multilevel vocational school-based intervention on physical activity and sedentary behavior: A cluster randomized trial)

***Cut-off for low vs. high MVPA stratification***

At the time of defining the protocol, we had not set a certain cut-off for the low vs. high MVPA stratification, as there was only scarce evidence regarding accelerometry-measured MVPA levels in this age group, both regarding prevalence and health effects of MVPA. A feasible cut-off point was judged to exclude no more than 20% of the trial cohort. The power analysis in the protocol was performed using this cut-off. Ihe internal pilot study indicated that about a fourth of the cohort reached 90 minutes of daily MVPA. PA guideline cut-off point would be 75 minutes of daily MVPA, but this would cut out a larger proportion of the trial cohort we thought to be feasible.

***Self-reported MVPA question***

Self-reported MVPA was measured with a question that is part of the NordPAQ measure, with participants asked to report on the past seven days (on how many days the respondent was active at least 30 minutes, response options 0-7). For reliability purposes, we decided not to use here the entire NordPAQ scale with the estimated total hours and minutes, as it was apparent that several respondents had misunderstood the question and reported amount of MVPA during all week and not average amount during a day of the past week. Before conducting the analyses of changes, we evaluated the baseline distributions of alternate MVPA self-report variables. The rationale for not pre-defining a particular self-report measure was that without adequate knowledge of how the respondents would respond to the questions, there was a risk of selecting an unsuitable variable (e.g. with excess skew or no variance) for the analyses.
