# Supplementary material for: Effectiveness of the Let’s Move It multi-level vocational school-based intervention on physical activity and sedentary behavior: a cluster randomized trial
Source: Ann Behav Med. 2025 May 27;59(1):kaaf023. doi: 10.1093/abm/kaaf023 (PMC12169330; doi:10.1093/abm/kaaf023)
Supplement: kaaf023_suppl_Supplementary_Files_3 [file kaaf023_suppl_supplementary_files_3.docx]

**Supplementary file 3. Fidelity of delivery of student session components in the Let’s Move It RCT intervention arm: Facilitator checklists (self-report)**

**S3 Table 1.** Percentage of components delivered fully as intended, partly or not delivered at all per session.

|  | Session 1 | Session 2 | Session 3 | Session 4 | Session 5 | Session 6 | Booster |
| --- | --- | --- | --- | --- | --- | --- | --- |
| Delivered fully as intended | 78.41 % | 66.67 % | 66.67 % | 71.11 % | 67.41 % | 75.80 % | 78.15 % |
| Partly delivered | 19.03 % | 28.17 % | 28.17 % | 25.56 % | 30.80 % | 18.80 % | 18.87 % |
| Not delivered | 2.56 % | 5.16 % | 5.16 % | 3.33 % | 1.79 % | 2.40 % | 2.98 % |

**S3 Table 2.** Omitted components, % of group sessions.

| **Session** |  | **Items not delivered** | **Omission percentage** |
| --- | --- | --- | --- |
| Session 1 | S1_4 | I conducted the Good group exercise | 3.33% |
|  | S1_6 | I showed the This Girl Can campaign video | 8.33% |
|  | S1_10 | I presented the LMI website, username and password to the students | 9.09% |
|  | S1_12 | I gave the intervention letter to guardians/parents to the students | 20.00% |
| Session 2 | S2_2 | I revisited the most important messages of the previous session | 25.00% |
|  | S2_9 | I revisited the most important messages of the current session | 21.43% |
| Session 3 | S3_7 | I provided the students discounts for physical activity experiments | 6.67% |
|  | S3_6 | I presented the LMI Facebook and Instagram pages to students | 18.18% |
|  | S3_9 | I revisited the most important messages of the session | 11.11% |
|  | S3_10 | Students filled the questionnaire after the session | 6.67% |
| Session 4 | S4_2 | I revisited the most important messages of the previous session | 3.57% |
|  | S4_4 | I presented, prompted and participated in the activity break | 3.57% |
|  | S4_8 | I revisited the most important questions of the current session | 7.14% |
| Session 5 | S5_5 | I conducted the Coping Plan Consultants exercise from the workbook | 3.45% |
|  | S5_6 | I revisited the most important ponderings of the session | 3.45% |
|  | S5_7 | I reminded the students about returning the workbooks* | 10.34% |
|  | S5_8 | I highlighted coping planning strategies | 5.00% |
| Session 6 | S6_1 | I took attendance* | 3.33% |
|  | S6_2 | I revisited the most important messages of the current session | 11.11% |
|  | S6_9 | I collected the workbooks* | 10.00% |
|  | S6_10 | I mentioned the LMI Facebook and Instagram accounts | 13.33% |
| Booster | S7_2 | I asked the students how they have been doing after Let's Move It | 4.00% |
|  | S7_6 | I revisited and presented the group-specific exemplary physical activity map | 12.00% |
|  | S7_9 | I mentioned the Facebook and Instagram accounts and Let's Move It website and the password | 8.00% |

*Not active ingredients of the intervention

**S3 Table 3**. Component delivery, % of group sessions. Full descriptions of content of the sessions can be found from Supplementary table 6 (<https://osf.io/ztbm3/>) for Hankonen et al. (2020).

| **Code** | **Component** | **Full delivery %** | **Partial delivery %** | **No delivery %** |
| --- | --- | --- | --- | --- |
| **Session 1** | **Start** |  |  |  |
| S1_1 | I took attendance* | 90.00 % | 10.00 % |  |
| S1_2 | I presented the LMI program | 80.00 % | 20.00 % |  |
| S1_3 | I conducted the Mood/Feeling Cards exercise | 93.33 % | 6.67 % |  |
| S1_4 | I conducted the Good Group exercise | 80.00 % | 16.67 % | 3.33 % |
| S1_5 | I conducted the Physical Activity Identity Continuum exercise | 44.83 % | 55.17 % |  |
| S1_6 | I showed the This Girl Can campaign video | 66.67 % | 25.00 % | 8.33 % |
| S1_7 | I conducted the Benefits of Physical Activity Cards exercise (for detecting personal motives) | 75.86 % | 24.14 % |  |
| S1_8 | I conducted discussions on the sitting statements | 80.00 % | 20.00 % |  |
| S1_9 | I presented instructions for using physical activity equipment | 77.27 % | 22.73 % |  |
| S1_10 | I presented the LMI website, username and password to students | 72.73 % | 18.18 % | 9.09 % |
| S1_11 | I gave the LMI workbooks to students | 93.33 % | 6.67 % |  |
| S1_12 | I gave the intervention letter for guardians/parents to the students | 80.00 % |  | 20.00 % |
| S1_13 | I revisited the most important messages of the session | 88.89 % | 11.11 % |  |
| S1_14 | I instructed the weekly PA Self-Monitoring Diary ACTION exercise | 70.00 % | 30.00 % |  |
| **Session 2** | **Get fit** | **Full delivery %** | **Partial delivery %** | **No delivery %** |
| S2_1 | I took attendance* | 100.00 % |  |  |
| S2_2 | I revisited the most important messages of the previous session | 60.71 % | 14.29 % | 25.00 % |
| S2_3 | I revisited the Good Group rules | 75.00 % | 25.00 % |  |
| S2_4 | I reviewed the Physical Activity Diary ACTION exercise | 46.43 % | 53.57 % |  |
| S2_5 | I conducted the Fitness knowledge quiz, round 1 | 85.71 % | 14.29 % |  |
| S2_6 | I conducted the Fitness knowledge quiz, round 2 | 64.29 % | 35.71 % |  |
| S2_7 | I raised the important messages in regards to becoming fit safely | 75.00 % | 25.00 % |  |
| S2_8 | I instructed the Incidental Physical Activity ACTION exercise | 60.71 % | 39.29 % |  |
| S2_9 | I revisited the most important messages of the session | 32.14 % | 46.43 % | 21.43 % |
| **Session 3** | **How and where?** | **Full delivery %** | **Partial delivery %** | **No delivery %** |
| S3_1 | I took attendance* | 90.00 % | 10.00 % |  |
| S3_2 | I revisited the previous session’s How Fit Am I? exercise | 68.97 % | 31.03 % |  |
| S3_3 | I reviewed the Incidental Physical Activity ACTION exercise | 40.00 % | 60.00 % |  |
| S3_4 | I conducted the Physical Activity Map exercise in groups of four | 90.00 % | 10.00 % |  |
| S3_5 | I instructed the Physical Activity Experiment ACTION exercise | 63.33 % | 36.67 % |  |
| S3_6 | I presented Let's Move It home training videos | 80.00 % | 20.00 % |  |
| S3_7 | I provided the students discounts for physical activity experiments | 66.67 % | 26.67 % | 6.67 % |
| S3_8 | I presented the LMI Facebook and Instagram pages to the students | 54.55 % | 27.27 % | 18.18 % |
| S3_9 | I revisited the most important messages of the current session | 77.78 % | 11.11 % | 11.11 % |
| S3_10 | Students filled the online questionnaire after the session | 80.00 % | 13.33 % | 6.67 % |
| **Session 4** | **Be smart** | **Full delivery %** | **Partial delivery %** | **No delivery %** |
| S4_1 | I took attendance* | 100.00 % |  |  |
| S4_2 | I revisited the most important messages of the previous session | 42.86 % | 53.57 % | 3.57 % |
| S4_3 | I reviewed the Physical Activity Experiment ACTION exercise | 42.86 % | 57.14 % |  |
| S4_4 | I presented, prompted and participated in the activity break | 60.71 % | 35.71 % | 3.57 % |
| S4_5 | I conducted the Critical assessment of popular myths in Fitspiration/Body Image Ideals exercise | 75.00 % | 25.00 % |  |
| S4_6 | I debriefed the Fitspiration/Body Image Exercise together with the whole group | 78.57 % | 21.43 % |  |
| S4_7 | I instructed the Personal SMART Goal and Plan ACTION exercise | 64.29 % | 35.71 % |  |
| S4_8 | I revisited the most important messages of the session | 75.00 % | 17.86 % | 7.14 % |
| **Session 5** | **Barriers on the way?** | **Full delivery %** | **Partial delivery %** | **No delivery %** |
| S5_1 | I took attendance* | 96.55 % | 3.45 % |  |
| S5_2 | I reviewed the Personal SMART Goal and Plan ACTION exercise | 31.03 % | 68.97 % |  |
| S5_3 | During the ACTION review, three ideas to gain support from others were presented by students or by me | 76.00 % | 24.00 % |  |
| S5_4 | I conducted the Barriers and solutions of PA exercise from the workbook | 72.41 % | 24.14 % | 3.45 % |
| S5_5 | I conducted the Coping Plan Consultants exercise in groups | 82.76 % | 17.24 % |  |
| S5_6 | I revisited the most important ponderings of this session | 75.86 % | 20.69 % | 3.45 % |
| S5_7 | I reminded the students about returning the workbooks* | 86.21 % | 3.45 % | 10.34 % |
| S5_8 | I highlighted coping planning strategies | 90.00 % | 5.00 % | 5.00 % |
| **Session 6** | **The journey continues** | **Full delivery %** | **Partial delivery %** | **No delivery %** |
| S6_1 | I took attendance* | 96.67 % |  | 3.33 % |
| S6_2 | I reviewed the Physical Activity Action Plan v.2 and Coping Planning ACTION exercise | 31.03 % | 68.97 % |  |
| S6_3 | I handed the students the sitting reduction checklist | 91.67 % | 8.33 % |  |
| S6_4 | I reviewed the 6 main messages of Let's Move It | 93.33 % | 6.67 % |  |
| S6_5 | I revisited the PA identity continuum exercise | 60.00 % | 40.00 % |  |
| S6_6 | I conducted the Behaviour Change Skills in Action exercise | 83.33 % | 16.67 % |  |
| S6_7 | I instructed the SMART Action Plan for 4 Weeks ACTION exercise | 70.00 % | 30.00 % |  |
| S6_8 | I revisited the most important messages of the session | 66.67 % | 22.22 % | 11.11 % |
| S6_9 | I collected the workbooks from the students* | 90.00 % |  | 10.00 % |
| S6_10 | I mentioned the Facebook and Instagram accounts | 73.33 % | 13.33 % | 13.33 % |
| S6_11 | I thanked the students for participating in the program | 96.67 % | 3.33 % |  |
| **Session 7** | **Booster session** | **Full delivery %** | **Partial delivery %** | **No delivery %** |
| S7_1 | I took attendance* | 96.00 % | 4.00 % |  |
| S7_2 | I asked the students how they have been doing after Let's Move It | 80.00 % | 16.00 % | 4.00 % |
| S7_3 | I revisited the Personally Important Reasons for PA exercise | 92.00 % | 8.00 % |  |
| S7_4 | I conducted the voting for favorite ways to reduce sitting from the Sitting Reduction Checklist | 80.00 % | 20.00 % |  |
| S7_5 | I revisited the most important points of the Physical Activity Identity Continuum exercise | 72.00 % | 28.00 % |  |
| S7_6 | I revisited and presented the group-specific exemplary physical activity map | 72.00 % | 16.00 % | 12.00 % |
| S7_7 | I conducted the Coping Plan Consultants problem solving exercise | 96.00 % | 4.00 % |  |
| S7_8 | I conducted the PA map and action plan for the spring/autumn ACTION exercise | 64.00 % | 36.00 % |  |
| S7_9 | I mentioned the Facebook and Instagram accounts and Let's Move It website and the password | 68.00 % | 24.00 % | 8.00 % |
| S7_10 | I thanked the students for participating in the LMI program | 100.00 % |  |  |

*Not active ingredients of the intervention

**References:**

Hankonen N, Absetz P, Araújo-Soares V. Changing activity behaviours in vocational school students: the stepwise development and optimised content of the ‘Let’s Move It’ intervention. Health Psychology and Behavioral Medicine. 2020;8:440–60.
