# Supplementary material for: Effectiveness of the Let’s Move It multi-level vocational school-based intervention on physical activity and sedentary behavior: a cluster randomized trial
Source: Ann Behav Med. 2025 May 27;59(1):kaaf023. doi: 10.1093/abm/kaaf023 (PMC12169330; doi:10.1093/abm/kaaf023)
Supplement: kaaf023_suppl_Supplementary_Files_4 [file kaaf023_suppl_supplementary_files_4.docx]

**Supplementary file 4 (S4). Supplementary tables**

for the manuscript Effectiveness of the Let’s Move It multilevel vocational school-based intervention on physical activity and sedentary behavior: A cluster randomized trial

[S4 Table 1. Initial analyses of intervention effects on activity outcomes (overall activity, not split to weekdays and weekend) 1](#_Toc72414389)

[S4 Table 2. Analyses of intervention effects on activity outcomes (not adjusted for parents' birth country, parents' education nor study track). 2](#_Toc72414390)

[S4 Table 3. Initial analyses of intervention effects on secondary outcomes. 3](#_Toc72414391)

[S4 Table 4. Analyses of intervention effects on secondary outcomes: lean and fat mass not adjusted for the season of measurement. 4](#_Toc72414392)

[S4 Table 5. Attendance differences in outcomes. Attendances above and below 50% compared to non-attendance (0%; incl. control group). 1](#_Toc72414393)

# S4 Table 1. Initial analyses of intervention effects on activity outcomes (overall activity, not split to weekdays and weekend)

|  | **Intervention** | | |  | **Control** | | |  | **Between groups** | | |
| --- | --- | --- | --- | --- | --- | --- | --- | --- | --- | --- | --- |
|  | **BL (SD)** | **Change, Mean (95% CI)** | |  | **BL (SD)** | **Change, Mean (95% CI)** | |  | **Treatment effect, B (95% CI)** | | **LMM*** |
| **Outcome** |  | **T3 - BL** | **T4 - BL** |  |  | **T3 - BL** | **T4 - BL** |  | **T3 - BL** | **T4 - BL** | **p-value** |
| Light PA, min/day | 172.1 (52.7) | 0.2  (-5.4–5.8) | -5.5  (-12.9–1.9) |  | 169.3 (51.9) | -6.1  (-11.0– -1.1) | 0.3  (-6.7–7.4) |  | 8.034  (1.435–14.634) | -1.541  (-11.003– 7.921) | 0.146 |
| MVPA, min/day | 68.0 (31.5) | -0.6  (-4.0–2.8) | -7.3  (-11.5– -3.1) |  | 63.3 (27.8) | 1.4  (-1.5–4.2) | -4.1  (-8.1– -0.1) |  | -0,394  (-4.427– 3.640) | -0.164  (-5.019– 4.692) | 0.667 |
| Steps | 8221.9 (3072.8) | 68.9  (-298.6– 436.4) | -614.4  (-1033.1– -195.7) |  | 7752.0 (2875.6) | 88.1  (-192.8– 369.0) | -329.1  (-737.1– 78.9) |  | 157.621  (-261.623– 576.865) | 23.269  (-467.352– 513.891) | 0.880 |
| Standing, min/day | 86.2 (40.6) | 0.3  (-3.3–3.8) | 0,4  (-6.0–6.9) |  | 82.5 (39.1) | 2.0 (-2.1–6.0) | 1.8 -(3.3–6.9) |  | 0.039  (-5.119– 5.196) | 1.073  (-5.964– 8.110) | 0.989 |
| SB | 534.8 (97.9) | -23.9  (-34.9– -13.0) | -14.9  (-29.5– -0.3) |  | 519.6 (108.4) | -8.4  (-19.5–2.8) | -4.5  (-18.8–9.8) |  | -1.660  (-12.934– 9.613) | 9.548  (-5.410– 8.110) | 0.653 |
| Breaks in SB | 27.0 (7.8) | -0.4  (-1.3–0.4) | -2.1  (-3.2– -1.0) |  | 24.8 (7.4) | 0.1  (-0.6–0.9) | 0.3  (-0.7–1.4) |  | 0.458  (-0.522– 1.439) | -0.486  (-1.796– 0.824) | 0.048 |

BL=Baseline; *Linear mixed model adjusted for gender and age. Classroom as random effect. The p value describes the difference between arms across the entire time period.

# S4 Table 2. Analyses of intervention effects on activity outcomes (not adjusted for parents' birth country, parents' education nor study track).

|  | **Intervention** | | | |  | **Control** | | |  | **Between groups** | | |
| --- | --- | --- | --- | --- | --- | --- | --- | --- | --- | --- | --- | --- |
|  | **BL mean (SD)** | **Change, Mean (95% CI)** | | |  | **BL mean (SD)** | **Change, Mean (95% CI)** | |  | **Treatment effect, B (95% CI)** | | **LMM*** |
| **Outcome** |  | **T3 - BL** | **T4 - BL** | |  |  | **T3 - BL** | **T4 - BL** |  | **T3 - BL** | **T4 - BL** | **p-value** |
| **Accelerometry, weekdays** | | | | | | | | | | | | |
| Light PA, min/day | 168.6 (51.5) | 1.9 (-3.7–7.4) | -4.4 (-11.7–3.0) |  | | 169.3 (51.7) | -5.0 (-9.8– -0.3) | -5.9 (-12.9–1.2) |  | 9.3 (2.8–15.9) | 4.6 (-4.3–13.4) | 0.01 |
| MVPA, min/day | 71.9 (31.0) | -0.7 (-4.3–3.0) | -6.8 (-11.1– -2.5) |  | | 68.0 (28.3) | 0.7 (-2.4–3.8) | -5.9 (-9.8– -2.0) |  | 0.2 (-4.1–4.5) | 1.4 (-3.4–6.3) | 0.85 |
| Steps | 8658.6 (3061.0) | 15.8  (-368.4–400) | -595.2  (-1031.9– -158.6) |  | | 8257.6 (2915.5) | 19.1  (-283.1–321.3) | -556.6  (-947.6– -165.7) |  | 199.3 (-237.4–636.1) | 209.9  (-276.4–696.3) | 0.76 |
| Standing, min/day | 88.5 (42.9) | -0.3 (-4.3–3.8) | -0.3 (-6.8–6.3) |  | | 86.3 (43.2) | 0.6 (-3.8–5.0) | -1.5 (-7.1–4.1) |  | 1.5 (-4.0–7.0) | 2.5 (-4.6–9.6) | 0.83 |
| SB | 543.9 (99.5) | -32.0  (-43.2– -20.8) | -23.8  (-37.1– -10.4) |  | | 522.9 (110.5) | -8.6 (-19.5–2.3) | -5.6 (-20.7–9.5) |  | -4.3 (-15.7–7.2) | 4.4 (-10.0–18.8) | 0.05 |
| Breaks in SB | 26.9 (7.6) | -0.4 (-1.2–0.5) | -1.9 (-3.0– -0.8) |  | | 24.9 (7.5) | 0.0 (-0.8–0.8) | -0.3 (-1.5–0.8) |  | 0.8 (-0.2–1.8) | 0.2 (-1.1–1.5) | 0.09 |
| **Accelerometry, weekend** | | | | | | | | | | | | |
| Light PA, min/day | 175.2 (81.2) | -8.5 (-20.6–3.6) | 0.6 (-14.6–15.8) |  | | 169.4 (77.1) | -12.1  (-22.6– -1.6) | 17.2 (3.0–31.5) |  | 4.6 (-8.4–17.7) | -2.0 (-20.5–16.6) | 0.76 |
| MVPA, min/day | 51.2 (41.7) | 0.4 (-6.0–6.8) | -7.3 (-14.8–0.3) |  | | 52.3 (42.8) | -2.3 (-8.7–4.0) | -1.3 (-9.5–7.0) |  | 1.9 (-5.9–9.7) | -2.9 (-11.3–5.4) | 0.20 |
| Steps | 6380.2 (4112.4) | 153.5  (-531.7–838.6) | -495.5  (-1275.8–284.9) |  | | 6530.3 (4400.5) | -222.8  (-839.5–394.0) | 61.9  (-816.1–940.0) |  | 298.2  (-493.4–1089.7) | -227.5  (-1125.9–670.8) | 0.19 |
| Standing, min/day | 76.7 (52.1) | -1.7 (-9.0–5.7) | 3.9 (-6.2–14.1) |  | | 72.5 (45.7) | 4.5 (-2,4–11.4) | 10.1 (1.1–19.1) |  | -5.0 (-14.5–4.6) | 1.7 (-9.7–13.1) | 0.74 |
| SB | 499.4 (146.3) | 0.3 (-23.8–24.5) | -3.9 (-34.2–26.5) |  | | 500.1 (152.4) | -5.9 (-28.3–16.6) | 10.9 (-14.2–35.9) |  | 3.6 (-18.1–25.3) | 6.9 (-22.2–36.1) | 0.69 |
| Breaks in SB | 26.3 (11.8) | -1.4 (-3.0–0.3) | -2.1 (-4.2–0.0) |  | | 24.5 (10.9) | -0.4 (-2,0–1.2) | 1.5 (-0.4–3.5) |  | -0.5 (-2.4–1.4) | -0.5 (-2.9–1.9) | 0.24 |
| **Accelerometry, school hours** | | | | | | | | | | | | |
| Light PA, min/day | 65.8 (22.3) | 9.4 (5.9–12.9) | 9.7 (4.8–14.5) |  | | 71.4 (29.3) | -3.7 (-7.5–0.1) | 0.8 (-5.0–6.7) |  | 11.2 (6.6–15.8) | 4.6 (-2.3–11.5) | <0.001 |
| MVPA, min/day | 35.1 (12.8) | 0.2 (-1.9–2.3) | 0.6 (-1.7–2.9) |  | | 31.3 (11.4) | 0.1 (-1.9–2.0) | 2.3 (-0.6–5.1) |  | 2.6 (0.1–5.1) | 2.9 (-0.3–6.2) | 0.62 |
| Steps | 4157.4 (1295.3) | 183.6  (-35.1–402.4) | 209.4  (-28.9–447.7) |  | | 3803.2 (1268.9) | 18.2  (-196.7–233.0) | 287.9 (-8.7–584.5) |  | 388.6  (116.2–661.2) | 335.8  (9.1–662.5) | 0.12 |
| Standing, min/day | 44.4 (25.0) | 2.6 (-0.3–5.5) | 2.2 (-2.0–6.5) |  | | 45.8 (27.7) | 0.3 (-3.6–4.2) | 1.2 (-3.5–5.8) |  | 2.4 (-2.0–6.8) | 1.4 (-3.8–6.5) | 0.31 |
| SB | 254.6 (40.3) | -18.2 (-24.3– -12.0) | -22.5 (-31.0– -14.1) |  | | 246.3 (54.3) | -4.0 (-11.8–3.7) | -10.9 (-21.9–0.1) |  | -13.6 (-21.9– -5.3) | -4.5 (-15.3–6.2) | 0.001 |
| Breaks in SB | 11.6 (3.4) | 1.1 (0.6–1.7) | 0.2 (-0.4–0.8) |  | | 11.0 (3.6) | 0.2 (-0.4–0.7) | 0.6 (-0.3–1.5) |  | 1.2 (0.5–1.8) | 0.2 (-0.7–1.0) | 0.12 |
|  | | | | | | | | | | | | |
|  |  |  |  |  | |  |  |  |  |  |  |  |

BL=Baseline; T3 = Time 3, 2-month follow-up; T4 = Time 4, 14 month. *Linear mixed model adjusted for gender and age. Classroom as random effect. The p value describes the difference between arms across the entire time period.

# S4 Table 3. Initial analyses of intervention effects on secondary outcomes.

|  | **Intervention** | | |  | **Control** | | |  | **Between groups** | | |
| --- | --- | --- | --- | --- | --- | --- | --- | --- | --- | --- | --- |
|  | **BL mean (SD)** | **Change, Mean (95% CI)** | |  | **BL mean (SD)** | **Change, Mean (95% CI)** | |  | **Treatment effect, B (95% CI)** | | **LMM*** |
| **Outcome** |  | **T3 - BL** | **T4 - BL** |  |  | **T3 - BL** | **T4 - BL** |  | **T3 - BL** | **T4 - BL** | **p-value** |
| Fat mass, kg | 16.96 (9.11) | N/A | 0.98  (0.60–1.37) |  | 17.75 (10.13) | N/A | 1.00  (0.062–1.37) |  | N/A | -0.004  (-0.542–0.534) | 0.99 |
| Lean mass, kg | 48.34 (10.00) | N/A | 1.32  (1.01–1.62) |  | 49.38 (10.05) | N/A | 1.84  (1.59–2.09) |  | N/A | -0.502  (-0.889– -0.115) | 0.006 |
| Self-reported MVPA (co-primary outcome) | 2.8 (1.9) | 0.26  (0.06–0.47) | 0.11  (-0.13–0.34) |  | 2.8 (1.9) | -0.03  (-0.24–0.18) | -0.21  (-0.43–0.01) |  | 0.096  (-0.146–0.338) | 0.119  (-0.153–0.391) | 0.11 |
| Injuries (7-day diary; %) | 9.2 | 2.0 (-2.3–6.4) | 1.6 (-3.5–6.6.) |  | 10.9 | -1.1 (-5.8–3.4) | -0.5 (-5.6–4.6) |  | 3.2 (1.0–5.4) | 2.1 (0.1–4.0) | 0.13 |
| Illnesses (7-day diary; %) | 28.5 | 1.9 (-4.9–8.8) | 3.9 (-4.5–12.4) |  | 30.2 | -4.1 (-11.2–3.0) | 4.9 (-3.0–12.7) |  | 6.1 (3.1–9.1) | -0.9 (-4.8–2.9) | 0.24 |
| Injuries (questionnaire; %) | 6.9 | 0.8 (-1.7–3.4) | 1.6 (-2.2–5.5) |  | 8.9 | -0.7 (-3.8–2.3) | 0.0 (-3.5–3.5) |  | 1.6 (0.4–2.7) | 1.6 (0.2–3.1) | 0.25 |
| Illnesses (questionnaire; %) | 6.5 | 2.1 (-0.9–5.2) | -1.0 (-4.6–2.6) |  | 6.4 | 1.4 (-1.9–4.8) | 3.6 (-0.2–7.4) |  | 0.7 (-1.0–2.4) | -4.6 (-6.8– -2.4) | 0.36 |
| Neck and shoulder pain symptoms; % | 56.2 | 2.7(-1.8–7.2) | 5.2 (-0.7–11.0) |  | 56.5 | 2.2 ( -2.2–6.6) | 4.1 (-1.2–9.4) |  | 0.5 (-1.6–2.6) | 1.1 (-2.2–4.4) | 0.68 |
| Lower back pain symptoms; % | 49.9 | 4.5 (-0.1–9.0) | 3.7 (-2.3–9.8) |  | 50.0 | -0.5 (2.9–7.0) | 0.3 (-5.5–6.1) |  | 5.0 (2.9–7.0) | 3.5 (1.2–5.7) | 0.94 |
| Headache symptoms; % | 62.1 | 3.6 (-0.7–7.9) | 2.8 (-3.0–8.5) |  | 59.8 | 7.2 (2.8–11.6) | 7.1 (2.0–12.2) |  | -3.6 (-6.7– -0.6) | -4.3 (-7.6– -1.0) | 0.80 |
| Self-reported fitness, poor; % | 47.5 | -0.6 (-4.9–3.6) | -2.6 (-7.9–2.6) |  | 43.9 | 2.1 (-2.5–3.6) | 8.1 (2.4–13.8.) |  | -2.8 (-4.3– -1.2) | -10.7 (-14.1–7.3) | 0.02 |

BL=Baseline; T3 = Time 3, 2-month follow-up; T4 = Time 4, 14 month. Note. Pain symptom frequencies ranging from once a month to almost daily
*Linear mixed model adjusted for gender and age. Classroom as random effect. The p value describes the difference between arms across the entire time period.

# S4 Table 4. Analyses of intervention effects on secondary outcomes: lean and fat mass not adjusted for the season of measurement.

|  | **Intervention** | | |  | **Control** | | |  | **Between groups** | | |
| --- | --- | --- | --- | --- | --- | --- | --- | --- | --- | --- | --- |
|  | **BL mean (SD)/%** | **Change, Mean (95% CI)** | |  | **BL mean (SD)/%** | **Change, Mean (95% CI)** | |  | **Treatment effect, B (95% CI)** | | **LMM*** |
| **Outcome** |  | **T3 - BL** | **T4 - BL** |  |  | **T3 - BL** | **T4 - BL** |  | **T3 - BL** | **T4 - BL** | **p-value** |
| Fat mass, kg | 16.96 (9.11) | N/A | 0.98 (0.60–1.37) |  | 17.75 (10.13) | N/A | 1.00  (0.062–1.37) |  | N/A | -0.136  (-0.765–0.492) | 0.67 |
| Lean mass, kg | 48.34 (10.00) | N/A | 1.32 (1.01–1.62) |  | 49.38 (10.05) | N/A | 1.84  (1.59–2.09) |  | N/A | -0.601  (-1.016– -0.185) | 0.005 |
| Injuries (7-day diary; %) | 9.2 | 2.0 (-2.3–6.4) | 1.6 (-3.5–6.6.) |  | 10.9 | -1.1 (-5.8–3.4) | -0.5 (-5.6–4.6) |  | 3.2 (1.0–5.4) | 2.1 (0.1–4.0) | 0.29 |
| Illnesses (7-day diary; %) | 28.5 | 1.9 (-4.9–8.8) | 3.9 (-4.5–12.4) |  | 30.2 | -4.1 (-11.2–3.0) | 4.9 (-3.0–12.7) |  | 6.1 (3.1–9.1) | -0.9 (-4.8–2.9) | 0.55 |
| Injuries (questionnaire; %) | 6.9 | 0.8 (-1.7–3.4) | 1.6 (-2.2–5.5) |  | 8.9 | -0.7 (-3.8–2.3) | 0.0 (-3.5–3.5) |  | 1.6 (0.4–2.7) | 1.6 (0.2–3.1) | 0.65 |
| Illnesses (questionnaire; %) | 6.5 | 2.1 (-0.9–5.2) | -1.0 (-4.6–2.6) |  | 6.4 | 1.4 (-1.9–4.8) | 3.6 (-0.2–7.4) |  | 0.7 (-1.0–2.4) | -4.6 (-6.8– -2.4) | 0.31 |
| Neck and shoulder pain symptoms; % | 56.2 | 2.7(-1.8–7.2) | 5.2 (-0.7–11.0) |  | 56.5 | 2.2 ( -2.2–6.6) | 4.1 (-1.2–9.4) |  | 0.5 (-1.6–2.6) | 1.1 (-2.2–4.4) | 0.63 |
| Lower back pain symptoms; % | 49.9 | 4.5 (-0.1–9.0) | 3.7 (-2.3–9.8) |  | 50.0 | -0.5 (2.9–7.0) | 0.3 (-5.5–6.1) |  | 5.0 (2.9–7.0) | 3.5 (1.2–5.7) | 0.59 |
| Headache symptoms; % | 62.1 | 3.6 (-0.7–7.9) | 2.8 (-3.0–8.5) |  | 59.8 | 7.2 (2.8–11.6) | 7.1 (2.0–12.2) |  | -3.6 (-6.7– -0.6) | -4.3 (-7.6– -1.0) | 0.73 |
| Self-reported fitness, poor; % | 47.5 | -0.6 (-4.9–3.6) | -2.6 (-7.9–2.6) |  | 43.9 | 2.1 (-2.5–3.6) | 8.1 (2.4–13.8.) |  | -2.8 (-4.3– -1.2) | -10.7 (-14.1–7.3) | 0.17 |

BL=Baseline; T3 = Time 3, 2-month follow-up; T4 = Time 4, 14 month. Note: Pain symptom frequencies ranging from once a month to almost daily

*Model adjusted for gender, age, parents' birth country, parents' education and study track. Classroom as random effect. The p value describes the difference between arms across the entire time period.

# S4 Table 5. Attendance differences in outcomes. Attendances of more and less than three sessions compared to non-attendance (incl. control group).

| **Outcome** | **LMM* for attendance of more than 3 sessions** | **LMM* for attendance less than 3 sessions** |
| --- | --- | --- |
| **Accelerometry, weekdays** |  |  |
| Light PA, min/day | 0.005 | 0.375 |
| MVPA, min/day | 0.429 | 0.518 |
| Steps | 0.593 | 0.824 |
| Standing, min/day | 0.891 | 0.769 |
| SB | 0.068 | 0.026 |
| Breaks in SB | 0.060 | 0.111 |

*Model adjusted for gender, age, parents' birth country, parents' education and study track. Classroom as random effect.
